# Coordinating expectations through central bank projections

**DOI:** 10.1007/s10683-020-09684-6

**Published:** 2020-11-24

**Authors:** Fatemeh Mokhtarzadeh, Luba Petersen

**Affiliations:** 1 Department of Economics, University of Victoria, 3800 Finnerty Road, Victoria, BC V8P 5C2 Canada; 2 Department of Economics, Simon Fraser University, 8888 University Drive, Burnaby, BC V5A 1S6 Canada

**Keywords:** Expectations, Monetary policy, Projections, Communication, Credibility, Laboratory experiment, Experimental macroeconomics, C9, D84, E52, E58

## Abstract

Central banks are increasingly communicating their economic outlook in an effort to manage the public and financial market participants’ expectations. We provide original causal evidence that the information communicated and the assumptions underlying a central bank’s projection can matter for expectation formation and aggregate stability. Using a between-subject design, we systematically vary the central bank’s projected forecasts in an experimental macroeconomy where subjects are incentivized to forecast the output gap and inflation. Without projections, subjects exhibit a wide range of heuristics, with the modal heuristic involving a significant backward-looking component. Ex-Ante Rational dual projections of the output gap and inflation significantly reduce the number of subjects’ using backward-looking heuristics and nudge expectations in the direction of the rational expectations equilibrium. Ex-Ante Rational interest rate projections are cognitively challenging to employ and have limited effects on the distribution of heuristics. Adaptive dual projections generate unintended inflation volatility by inducing boundedly-rational forecasters to employ the projection and model-consistent forecasters to utilize the projection as a proxy for aggregate expectations. All projections reduce output gap disagreement but increase inflation disagreement. Central bank credibility is significantly diminished when the central bank makes larger forecast errors when communicating a relatively more complex projection. Our findings suggest that inflation-targeting central banks should strategically ignore agents’ irrationalities when constructing their projections and communicate easy-to-process information.

**Electronic supplementary material:**

The online version of this article (10.1007/s10683-020-09684-6) contains supplementary material, which is available to authorized users.

## Introduction

The economy is highly complex with many moving parts. It can be very challenging for the average person, with limited cognitive capacity and attention, to accurately forecast how it will evolve. To ease this cognitive burden, guide expectations, and improve monetary policy efficacy, which operates largely through an expectations channel, central banks have become increasingly transparent about their objectives, future policies, and their outlook on the future. Many central banks publish a combination of projections about future GDP, GDP growth, CPI, and their policy rates. The Reserve Banks of Australia, New Zealand, and Norges Bank were pioneers in the publication of their inflation projections during the early 1990s. Likewise, the Reserve Bank of New Zealand has communicated their projections for the 90-day bank bill rate via Monetary Policy Statements since 1997. Other central banks followed suit with Norway (2005), Sweden (2007), and the FOMC (2012) publishing their projections of key policy rates.

Central banks face two critical decisions when constructing and communicating their projections. First, they must make numerous assumptions about how the economy evolves, including how people think about the future. Many central banks construct their projections under the assumption that households and firms form rational expectations. While projections based on the assumption of non-rational expectations may be more accurate and may enhance central bank credibility, it is not clear which of many models of non-rational expectations to use. Assumptions about the form of aggregate expectations can have important implications for predicted aggregate dynamics and optimal monetary policy. Moreover, the information in projections has the potential to influence the heuristics individuals use to form their expectations.^1^ Second, central banks must decide which of their many projections to communicate to the public. Too little information may insufficiently guide public expectations and the central bank to be perceived as “opaque”; too much information can result in cognitive overload and audiences not paying sufficient attention to any particular information.

The contribution of this paper is to provide empirical insight into these two important policy decisions. We study individual and aggregate forecasts in 24 multi-period laboratory economies where we can systematically control the information that central banks communicate about their projections in otherwise identical underlying economies. In each period of our experiments, each subject reports incentivized forecasts of the following period’s rate of inflation and the output gap. Aggregate expectations and a random disturbance to aggregate demand endogenously determine the current state of the economy.

We study the effects of four different types of central bank projections on individual forecasting heuristics and aggregate dynamics. In our baseline environment, participants observe current and historical information about the economy, as well as full information about the economy’s data-generating process. We compare our benchmark economies, where the central bank does not communicate its projections, to treatment economies operating under three alternative communication policies. In our Interest Rate Projection treatment, all subjects observe the central bank’s projection of future nominal interest rates, derived according to the economy’s rational expectations equilibrium (REE) solution. In the Dual Projections treatment, all subjects are instead informed about the central bank’s projection of future inflation and the output gap, also derived using the REE solution. For a rational subject, the communications in either of these two projection treatments are redundant and should not influence expectations. For boundedly rational subjects, however, such projections provide potentially useful focal information. While both of these REE projections convey the same overall information about the economy, we hypothesized that Dual Projections would be cognitively less demanding for subjects to apply to their private output gap and inflation forecasts. Finally, the Adaptive Dual Projections treatment mirrors the Dual Projections treatment except that the central bank’s projections follow an adaptive model of expectations that, based on previous work, we expect would better predict aggregate dynamics, and thus, reduce credibility concerns. The purpose of the Adaptive Dual Projections treatment is to address discussions in policy circles as to whether to implement boundedly rational agents into central banks’ forecasting models.

We find that certain central bank projections can significantly stabilize expectations and the aggregate experimental economy by *nudging* naïve forecasters towards fundamentally-driven rational expectations. Rational projections of future output gap and inflation result in consistently less dispersion in forecasts and significantly smaller forecast errors. By contrast, projections of nominal interest rates lead to mixed results. For relatively low variability in aggregate demand shocks, subjects are willing and able to employ the projections, resulting in significantly more rational forecasts. However, as the variability of shocks increases, the ease and value of using the projection decreases, and subjects instead rely on adaptive forecasting heuristics. These results suggest that policymakers cannot take for granted that private agents can infer the implied path of inflation and the output gap from an interest rate projection. Rather, central banks concerned about anchoring a specific type of expectation should directly communicate about that variable of interest.

Adaptive dual projections generate significantly larger inflation forecast errors, greater forecast dispersion, and increased inflation variability. These effects are a consequence of a large fraction of subjects directly employing the central bank’s adaptive projections as their own while other participants respond to their counterparts’ adaptive behavior by forecasting even higher inflation. Our paper provides original empirical evidence that inflation-targeting central banks may prefer to avoid communicating a projection based on adaptive expectations.

Loss of credibility is a crucial concern central banks face when deciding whether to communicate their projections. We find that this concern is valid when the central bank communicates either a nominal interest rate projection or an adaptive dual projection. Under both types of projections, the likelihood a subject employs the central bank projection decreases as the central bank makes larger forecast errors in the recent past. Effective usage of the interest rate projections is very low as it is more challenging to infer what the projection implies about future output and inflation. In the Adaptive Dual Projections treatment, subjects’ credibility in the projection is very high but sensitive to the central bank’s forecast errors. As the central bank’s implied forecast of future output and inflation becomes increasingly incorrect, the likelihood that subjects utilize the projections significantly decreases. By contrast, the central bank’s credibility appears to be impervious to its forecast errors when it publishes rational dual projections.

Our paper complements the existing empirical and theoretical work on the role of central bank communication and projections in shaping expectations. The empirical literature has found mixed evidence on the effectiveness of forward guidance in influencing expectations (Kool and Thornton 2015; McCaw and Ranchhod 2002; Goodhart and Lim 2011; Brubakk et al. 2017; Turner 2006) while macroeconomic projections appear to more consistently manage inflation expectations. Hubert (2014) finds a significant positive relationship between inflation projections and forecasters’ expectations of inflation in Sweden, the UK, Canada, Japan, and Switzerland. In a closely related paper to ours, Jain and Sutherland (2018) construct an original panel data set of 23 countries to estimate the effects of numerous central bank projections and forward guidance on private-sector forecast dispersion and accuracy. They find that macroeconomic central bank projections are generally ineffective at reducing private-sector forecast dispersion and forecast errors about inflation and output growth. They do, however, reduce forecast errors and dispersion about short-term interest rates. Interest rate projections do not have a large or consistent effect on inflation expectations.

Goy et al. (2020) computationally examine agents’ expectations near and at the zero lower bound (ZLB). They find that forward guidance in the form of output and inflation projections significantly reduce the likelihood of deflationary spirals when the economy is at the ZLB. Likewise, theoretical work by Ferrero and Secchi (2010) highlights that macroeconomic projections are more effective than interest rate projections at stabilizing expectations of recursively learning agents. Our paper provides experimental validation of these results and additional insight into the consequences of modifying projections in response to the public’s backward-looking behavior. Moreover, our findings provide original empirical support for the policy recommendation that strict inflation-targeting central banks disregard the public’s adaptive forecasting heuristics when designing their communication strategy.

Learning-to-forecast experiments (LtFEs) are used to study how macroeconomic expectations respond to information, policy, and structural features of the economy. In LtFEs, subjects play the roles of forecasters and are tasked with forming accurate forecasts for the following period(s) over a long multi-period horizon. Each period, aggregated forecasts are used by computerized households, firms, and banks to make decisions according to a pre-specified data-generating process. In other words, subject-provided aggregate expectations about the future have a direct effect on the current macroeconomy. The assumption that expectations influence economic decision making is supported by recent experimental evidence. In a field experiment involving participants in the University of Michigan Survey of Consumers and RAND’s American Life Panel, Armantier et al. (2015) observe, on average, a correlation between participants’ expectations and decisions in a manner consistent with economic theory. Cornand and Hubert (2020) investigate the external validity of expectations elicited in LtFEs. They find that expectations elicited from undergraduate participants in LtFEs are consistent with those formed by households, firms, professional forecasters, financial market participants, and central banks in that forecast errors are large, serially correlated, and predictable indicative of information frictions.^2^

There are a handful of LtFEs that investigate the effect of central bank communication on expectation formation.^3^ Kryvtsov and Petersen (2013, 2020) study, among many things, the effects of central bank projections of nominal interest rates. They find that focal five-period ahead interest rate projections have an inconsistent effect on forecasting behavior. Many inexperienced subjects incorporate the projections into their forecast, leading to improved inflation stability in some sessions. However, if only a few subjects initially employ the projections in their forecasts, the announcement creates confusion, and expectations become increasingly destabilized. Like Kryvtsov and Petersen (2013), we find that nominal interest rate projections lead to heterogeneous heuristics. Our paper extends their findings by providing a more robust study of different types of projections. We additionally consider rationally- and adaptively-formed inflation and output gap projections to gain insight into the ability of central bank projections to influence expectations and maintain central bank credibility. The communication of inflation targets has also been shown to have mixed effects on the management of expectations. Under a dual mandate to stabilize both inflation and output gap, Cornand and M’baye (2018) find that expectations and inflation are better anchored, while Mirdamadi and Petersen (2018) observe increased heterogeneity in heuristics as forecasters have more information to coordinate on. Arifovic and Petersen (2017) find that communicating a time-varying, history-dependent inflation target can make expectations even more pessimistic at the ZLB when the central bank fails to achieve its explicit targets. Ahrens et al. (2016) have extended our paper and Arifovic and Petersen (2017) to study the effects of one-period ahead inflation projections in the presence of both demand and supply shock in the normal times or at the zero lower bound. Similar to our findings, they observe that central bank inflation projections significantly alter how subjects forecast and reduce economic instability at the zero lower bound.

The paper is organized as follows. Section 2 lays out our theoretical framework and experimental design. Results are presented in Sects. 3 and 4 discusses our findings in the context of the learning and inattention literature.

## Experimental design

Our experiment explores the formation of macroeconomic expectations in the presence of central bank projections of key macroeconomic variables. Each independent economy involves groups of seven inexperienced subjects tasked with playing the role of forecasters that submit incentivized forecasts about two evolving variables: the subsequent period’s output gap, $x_{t+1}$, and inflation, $\pi _{t+1}$. The submitted forecasts are aggregated as $\mathbb{E}_{t}^{*} x_{{t + 1}}$ and $\mathbb{E}_{t}^{*} \pi _{{t + 1}}$ and used by computerized households and firms to form optimal decisions, which in turn determines concurrent inflation and the output gap.

### Model

We designed our experiment around a simplified version of a rational expectations representative agent New Keynesian macroeconomic model. We focus on this specific model for its relative simplicity, because of its ubiquitous use by central banks over the last decade, and the vital role expectations play in driving aggregate dynamics. The aggregate economy implemented in our experiment is described by the following system of equations:^4^

1 $$x_{t} = \mathbb{E}_{t}^{*} x_{{t + 1}} - \sigma ^{{ - 1}} (i_{t} - \mathbb{E}_{t}^{*} \pi _{{t + 1}} - r_{t}^{n} ),$$

2 $$\pi _{t} = \beta \mathbb{E}_{t}^{*} \pi _{{t + 1}} + kx_{t}$$

3 $$\begin{aligned}&i_{t}= \phi _{\pi }\pi _{t}+\phi _{x}x_{t}, \end{aligned}$$

4 $$\begin{aligned}&r^{n}_{t} = \rho _{r}r^{n}_{t-1}+\epsilon _{rt}. \end{aligned}$$

Equation (1) is the Investment-Saving curve and describes the evolution of the output gap or aggregate demand. It is derived from a log-linear approximation of households’ intertemporal optimization around a deterministic zero inflation and output gap steady states. Equation (1) describes how the current output gap, $x_{t}$, depends positively on aggregated expectations of next period’s output gap, $\mathbb{E}_{t}^{*} x_{{t + 1}}$, and deviations of the real interest rate, $i_{t} - \mathbb{E}_{t}^{*} \pi _{{t + 1}}$, from the natural rate of interest, $r^{n}_{t}$.^5^ The quantitative importance of this deviation depends on the elasticity of intertemporal substitution, $\sigma ^{-1}$.

Equation (2) is the New Keynesian Phillips curve (NKPC) which describes the evolution of inflation, $\pi _{t}$, in response to changes in aggregated expectations of future inflation, $\mathbb{E}_{t}^{*} \pi _{{t + 1}}$, and the output gap, $x_{t}$. The coefficient $\kappa$ is a function of parameters associated with the frequency and the size of firms’ price changes and governs the sensitivity of prices to aggregate demand. The coefficient $\beta$ represents the subjective discount rate. To construct the NKPC, we make the simplifying assumption that households have identical information sets and form expectations using identical functions of the state history.

Equation (3) is the central bank’s policy rule and describes the evolution of the nominal interest rate. Under this specification the central bank contemporaneously responds to deviations of the output gap and inflation from their steady state values. In each period, the automated central bank increases the nominal interest rate in response to higher current inflation and the output gap. The coefficients $\phi _{\pi }$ and $\phi _{x}$ govern the central bank’s reaction to inflation and the output gap.^6^ Note that the implemented environment studies deviations around a constant steady state, ignoring the presence of zero lower bound. That is, $i_{t}$ was frequently negative in our experiment.^7^

Finally, Eq. (4) describes how the natural rate of interest evolves in response to random perturbations. Throughout the paper, we will refer to $r_{t}^{n}$ as a *shock* to the demand side of the economy that follows an *AR(1)* process. The random innovation, $\epsilon _{rt}$, is drawn from an *i.i.d *$N(0,\sigma _{r})$. The experimental economy’s data-generating process is calibrated to match moments of the Canadian data following Kryvtsov and Petersen (2013); $\sigma =1$, $\beta =0.989$, $\kappa =0.13$, $\phi _{\pi }=1.5$, $\phi _x=0.5$, $\rho _{r}=0.57$, and $\sigma _r=113$ basis points (bps). The environment has a unique steady state where $\pi ^{*}=x^{*}=i^{*}=r^{n*}=0$.

### Experimental implementation

A total of 168 undergraduate students participated in the experiment at the CRABE lab at Simon Fraser University from June 2015 to December 2016. Subjects were invited to participate in a single session from an inexperienced subject pool consisting of over 2000 subjects from a wide variety of disciplines. For each of our four treatments, we collected data from six groups of seven subjects each, for a total of 24 independent observations. Subjects participated in two 30-period repetitions with the same group. We describe subjects in Repetition 1 as *inexperienced* and Repetition 2 as *experienced*. Thus, we have a total of 10,080 observations. The experiment lasted for approximately 90 minutes, including 35 minutes of instruction.

Before the experiment began, participants received information about their forecasting task, how their forecast accuracy would determine their payoffs, and how the economy evolved in response to their expectations and aggregate shocks. They were provided with Eqs. (1)–(4), which were also explained qualitatively and intuitively in the instructions. We provided subjects with detailed quantitative information about the data-generating process to enable them to forecast accurately.^8^ Subjects were familiarized with the forecasting task with four unpaid trial periods. Subjects had the opportunity to ask questions about the data-generating process and their tasks privately.^9^

**Fig. 1:**
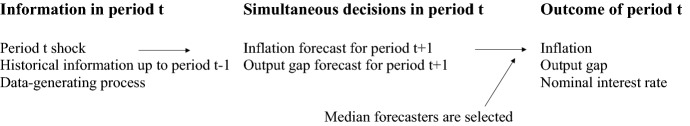
Timing of information, decisions, and outcomes in each round

**Fig. 2:**
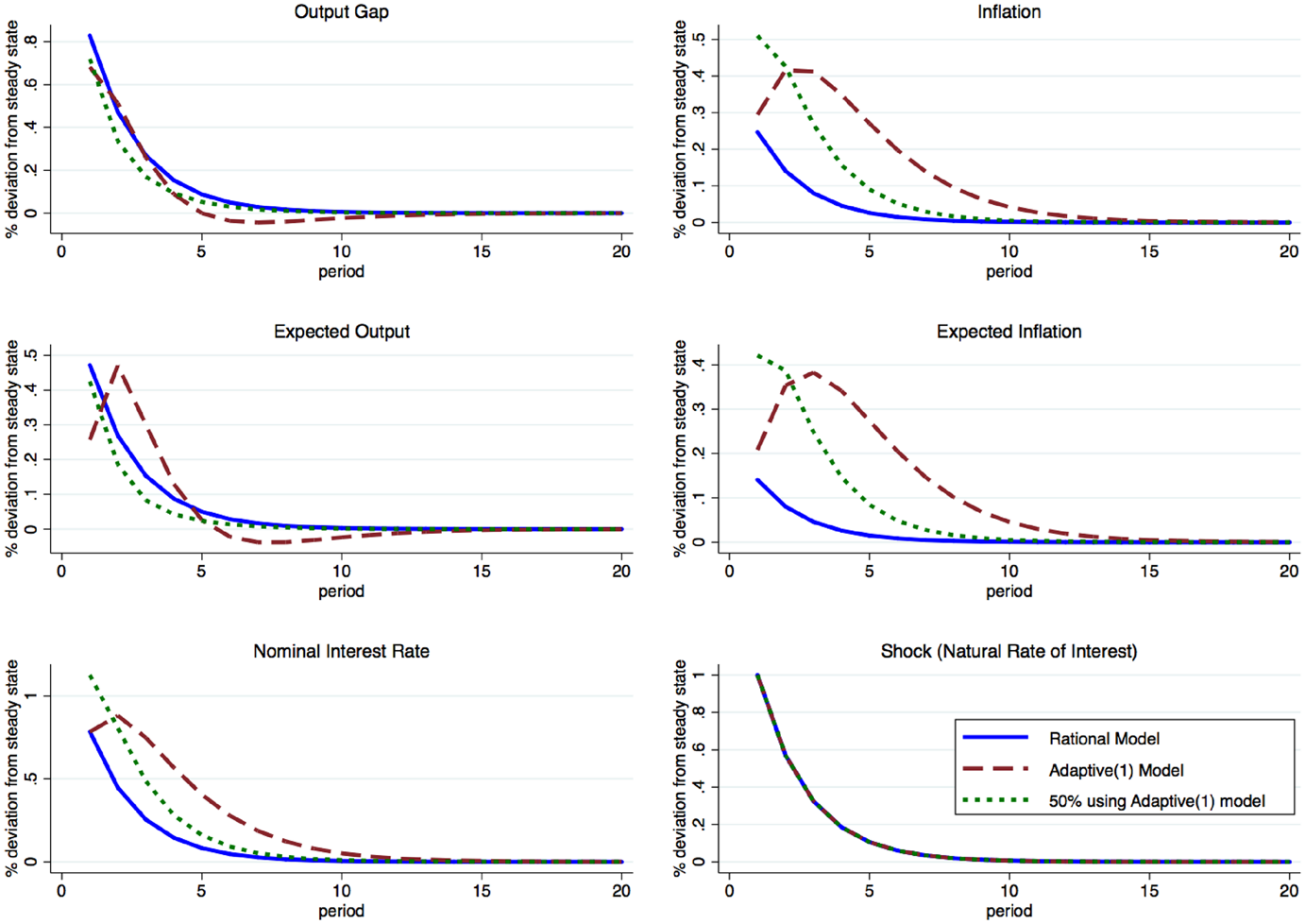
Simulated impulse responses to a 1 s.d. innovation to $r_{t}^{n}$ under alternative forecasting assumptions

**Fig. 3:**
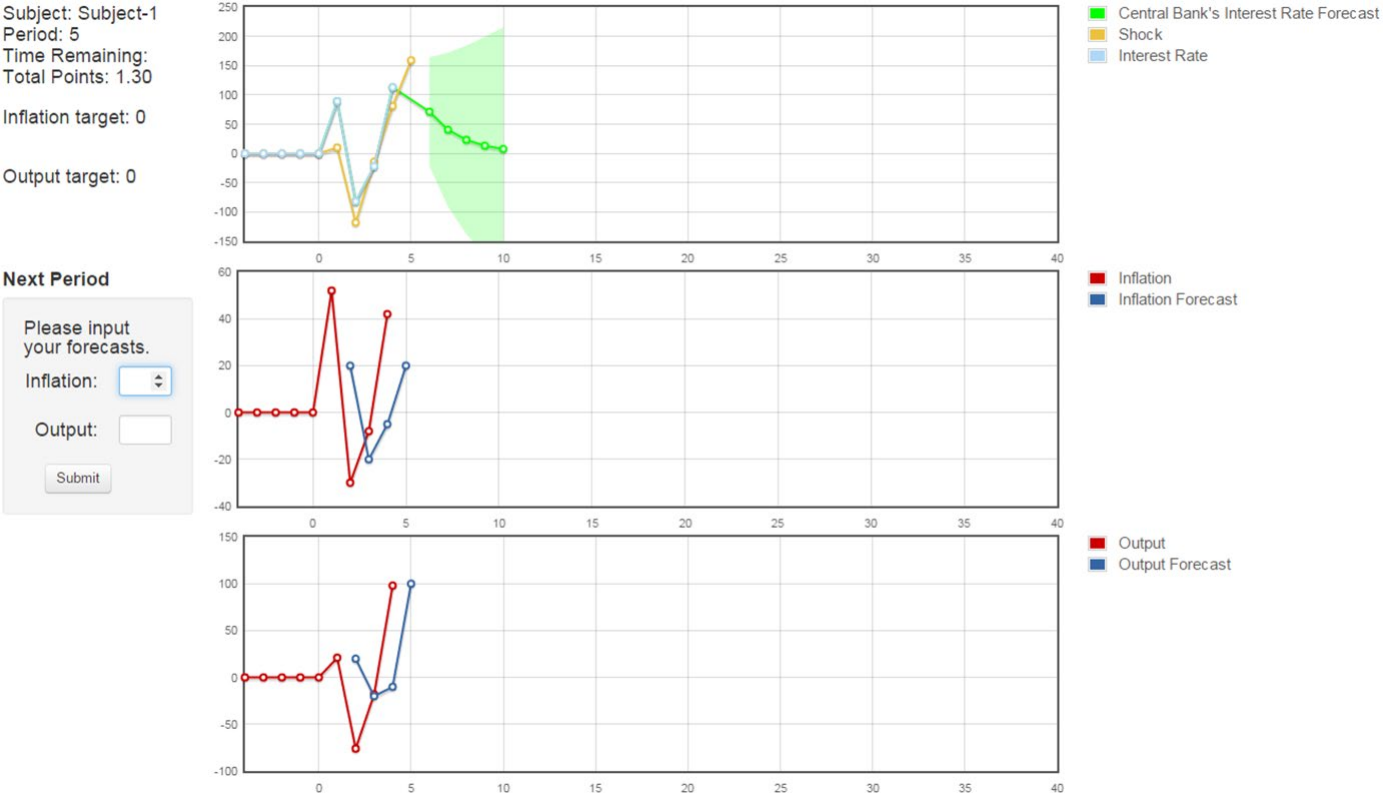
Screenshot from IRProj treatment. The figure presents a representative screenshot of the interface in the IRProj treatment with interest rate projections. In each period subjects are shown their identification number, current period, time remaining, and the total number of points earned along with the in ation and output gap targets. The top history panel is past interest rates, past and current shocks, and central bank projections of five-period ahead interest rate-green line- . The second panel is the subject's past forecasts of in ation and realized in ation and the third panel is the subject's forecasts and realized output gap

Figure 1 presents the timeline of decisions in the experiment. Before forming their forecasts at the start of a period, subjects had access to the following common information (and all subjects understood that this was common information). During the experiment, subjects observed all historical information up to and including the previous period’s realized inflation, output gap, nominal interest rate, and shocks, as well as their private forecasts (but not the history of other subjects’ forecasts or the aggregate forecasts). They also observed the current period shock, which allowed them to calculate the expected future shocks for the following periods. Forecasts were submitted in basis point measurements and could be positive, zero, or negative.

Aggregate expectations in the standard New Keynesian model are assumed to be homogeneous and rational across the representative household and continuum of firms. There is a need to compute aggregate expectations when we move to an experimental setting with groups of participants with potentially heterogeneous expectations. While the theory is silent on this, there are two natural approaches to computing aggregate expectations that feed into these DGPs: either take the mean or the median forecasts in a given period. With a relatively small sample of subjects per session, a median forecast is a better measure of central tendency than the average and avoids the effects of outlier forecasts. After all subjects privately submitted their forecasts or time elapsed, the median submitted forecasts for the output gap and inflation were employed as the aggregate forecasts and used to calculate the current period’s output gap, inflation, and nominal interest rate.^10^

We incentivized subjects to take seriously their forecasting decisions by rewarding them based on the accuracy of their forecasts. We follow Kryvtsov and Petersen (2013, 2020) by paying participants according to an exponential payoff function. Subject *i*’s earned points in period *t* was a function of her absolute inflation and output gap forecast errors in period $t-1$:

5 $$\begin{aligned} Score_{i,t}=0.3(2^{-0.01|E_{i,t-1}\pi _{i,t}-\pi _{t}|}+2^{-0.01|E_{i,t-1}x_{i,t}-x_{t}|}) \ , \end{aligned}$$

where $$|E_{i,t-1}\pi _{i,t}-\pi _{t}|$$ and $$|E_{i,t-1}x_{i,t}-x_{t}|$$ are subject *i*’s forecast errors associated with forecasts submitted in period $$t-1$$ for period *t* variables. For every 100 basis point error made for each of inflation and the output gap, a subject’s score would decrease by 50%. Thus, there was a strong incentive to forecast accurately. The per-period points ranged from 0 to 0.6 points. At the end of the experiment, subjects’ points from all periods were converted into dollars and paid out to them in cash. The maximum possible earnings, including a CDN $7 show-up fee, was $25. The average payment was CDN $19 and ranged from CDN $17 to $25.

The median forecasts formed in period *t* about period $t+1$ are used to determine the state of the economy in period *t*. The usage of the median ensures that any one participant cannot notably influence the aggregate expectations as one of only 7 subjects unless other subjects’ forecasts are widely dispersed. Given that participants do not know others’ forecasts or whether they are likely to be the median forecaster, we believe that subjects are well-incentivized to forecast accurately about the future. Further discussion of how this design decision interacts with the incentive scheme can be found in Section B of the Online Appendix. Further discussion of how this design decision interacts with the incentive scheme can be found in Section B of the Online Appendix.

Subjects participated in an online interface. Figure 3 presents a representative screenshot of the interface in the IRProj treatment with interest rate projections. The top left corner of the screen showed the subject’s identification number, current period, time remaining, and the total number of points earned. Three history panels were provided on the right side of the screen. The top history panel displayed past interest rates and past and current shocks. The second panel displayed the subject’s past forecasts of inflation and realized inflation. The final panel showed the subject’s forecasts and realized output gap. In treatments with central bank communication, additional time series were added to the history plots to represent the central bank’s projections. The central bank communicated its projections of inflation, the output gap, and nominal interest rates with green lines representing the expected future path of the respective variable. Around each projection was a one standard deviation confidence interval that increased as the projection went further into the future to reinforce that the central bank’s projections were noisy predictions.^11^

### Treatments

To investigate the impact of central bank projections on forecasting heuristics and economic stability, we systematically vary the type of projections subjects receive in a between-subject experimental design. A summary of our treatments is presented in Table 1.^12^

**Table 1:** Summary of treatments

Treatment	Sessions	Repetitions per session	Periods per repetition	Subjects per session	CB projected variables $s=1, \ldots ,5$	CB assumption on aggregate expectations
NoComm	6	2	30	7	None	None
IRProj	6	2	30	7	$i_{t+s}$	Rational
DualProj	6	2	30	7	$x_{t+s}$, $\pi _{t+s}$	Rational
ADProj	6	2	30	7	$x_{t+s}$, $\pi _{t+s}$	Adaptive(1)
*Aggregate statistics under alternative heuristics*						
	Rational		Adaptive(1)		ADProj	
SD *x*	101.6 bps		91.2 bps		84.4 bps	
SD $\pi$	30.3 bps		84.9 bps		91.8 bps	
Mean abs. forecast error *x*	0 bps		33.4 bps		18.2 bps	
Mean abs. forecast error $\pi$	0 bps		21.1 bps		20.2 bps	

Statistics are calculated based on Dynare simulations of 10,000 draws of innovations to the natural rate of interest, $r_{t}^n$, under different assumptions of aggregate expectations. Our hypotheses are based on the assumptions that subjects in NoComm employ the Adaptive(1) heuristic, in IRProj and DualProj the Rational heuristic, and in ADProj the ADProj heuristic

We conducted four treatments involving different types of communication. In all of our treatments, the central bank had the same complete information about the economy’s data-generating process as participants, namely Eqs. (1) to (4). The treatments differ in terms of the variables presented to participants and the assumptions underlying the central bank’s projections about aggregate expectations. Importantly, in all communication treatments, the central bank assumes that the median agents maintain the same heuristics in response to the economy. i.e., agents do not update the parameters of their forecasting model as new information and projections arrive.

Our baseline environment, *No Communication (NoComm)*, follows the experimental design described above with no supplementary communication by the central bank.

**Table 2:** Forecasting heuristics

Model	Heuristic name	Model
M1	Ex-Ante Rational	$E_{i,t}x_{t+1}=0.269r_{t-1}^n + 0.472\epsilon _{t}$
		$$E_{i,t}\pi _{t+1}=0.08r_{t-1}^n + 0.141\epsilon _{t}$$
M2	Adaptive(1)	$E_{i,t}x_{t+1}=0.146r_{t-1}^n + 0.536x_{t-1}-0.138\pi _{t-1}+0.257\epsilon _{t}$
		$$E_{i,t}\pi _{t+1}=0.119r_{t-1}^n + 0.037x_{t-1}+0.711\pi _{t-1}+0.208\epsilon _{t}$$
M3	ADProj	$E_{i,t}x_{t+1}=0.293r_{t-1}^n + 0.071x_{t-1}-0.276\pi _{t-1}+0.513\epsilon _{t}$
		$$E_{i,t}\pi _{t+1}=0.237r_{t-1}^n + 0.074x_{t-1}+0.422\pi _{t-1}+0.416\epsilon _{t}$$
M4	Best-response to ADProj	$E_{i,t}x_{t+1}=0.178r_{t-1}^n -0.021x_{t-1}-0.114 \pi _{t-1}+ 0.312\epsilon _{t}$
		$$E_{i,t}\pi _{t+1}=0.311r_{t-1}^n +0.031x_{t-1}+ 0.123\pi _{t-1}+ 0.546\epsilon _{t}$$
M5	Target	$E_{i,t}x_{t+1}=0$
		$$E_{i,t}\pi _{t+1}=0$$
M6	Naive	$E_{i,t}x_{t+1}=x_{t-1}$
		$$E_{i,t}\pi _{t+1}=\pi _{t-1}$$
M7	Constant gain	$E_{i,t}x_{t+1}=E_{t-1}x_{t}-\gamma (E_{i,t-2}x_{t-1}-x_{t-1})$
		$$E_{i,t}\pi _{t+1}=E_{t-1}\pi _{t}-\gamma (E_{i,t-2}\pi _{t-1}-\pi _{t-1})$$
M8	Trend chasing	$E_{i,t}x_{t+1}=x_{t-1}+\tau (x_{t-1}-x_{t-2})$
		$$E_{i,t}\pi _{t+1}=\pi _{t-1}+\tau (\pi _{t-1}-\pi _{t-2})$$

Models of expectations as functions of exogenous or historical data. $\gamma$ and $\tau \in \left[ 0.1,1.5\right]$ in increments of 0.1

In the *Interest Rate Projections (IRProj)* and *Dual Projections (DualProj)* treatments, the central bank communicated evolving five-period ahead projections of the nominal interest rate and dual projections of output gap and inflation, respectively. In both treatments, the central bank assumed the median agents formed their expectations according to the unique stationary rational expectations equilibrium (REE) solution. That is, the central bank assumed the median agents’ expectations were “model consistent” or “Ex-Ante Rational” as described by M1 in Table 2. Given its assumption of Ex-Ante Rational expectations, the central bank’s projected values, in period *t*, about period $t+s$ are given by

6 $$\begin{aligned} E_{t}^{CB}x_{t+s}&=\rho ^{s-1} \cdot (0.47 \cdot r^{n}_{t-1}+0.83 \cdot \epsilon _t),\nonumber \\ E_{t}^{CB}\pi _{t+s}&= \rho ^{s-1} \cdot ( 0.14 \cdot r^{n}_{t-1}+ 0.25 \cdot \epsilon _t), \nonumber \\ E_{t}^{CB}i_{t+s}&= \rho ^{s-1} \cdot (0.45 \cdot r^{n}_{t-1}+ 0.78 \cdot \epsilon _t) \end{aligned}$$

for $s=1, \ldots ,5$ and $\rho =0.57$. That is, the projected variables monotonically reverted back toward the steady state. If the median forecasters were to forecast according to the central bank’s explicit or implicit projections, the payoff-maximizing strategy of all other subjects would be to do the same. A subject’s optimal forecasts given the median forecasters’ usage of the central bank’s projections is given by Eq. (6).

Our fourth treatment, *Adaptive Dual Projections (ADProj)*, also involved providing subjects with five-period ahead projections of the output gap and inflation. In this treatment, the central bank instead assumed that the median forecasters formed output gap and inflation expectations as an equally-weighted average of a one-period lag of the output or inflation and the ex-post rational expectations solution, described by M3 in Table 2. This assumption is motivated by the findings of Kryvtsov and Petersen (2013) that such an Adaptive(1) forecasting heuristic well describes the median subjects’ forecasting heuristic. Such aggregate expectations would generate central bank forecasts for the output gap and inflation at time $t+s$:

7 $$\begin{aligned}&E_{t}^{CB}x_{t+s}=0.293r_{t-1}^n + 0.071x_{t-1}-0.276\pi _{t-1}+0.513\epsilon _{t} \end{aligned}$$

8 $$\begin{aligned}&E_{t}^{CB}\pi _{t+s}=0.237r_{t-1}^n + 0.074x_{t-1}+0.422\pi _{t-1}+0.416\epsilon _{t} \end{aligned}$$

The instructions informed subjects in the IRProj and DualProj treatments that the central bank projections were formed based on current and expected future shocks as well as the economy’s data-generating process. The ADProj instructions made it clear that the central bank constructed its projections using a combination of current and expected future shocks and the previous period’s outcomes. The quantitative models behind the projections were not provided to reduce information overload. We emphasized that the projections were not a promise but only the central bank’s forecast of the future, incorporating all available information. We did not provide any explanation for why the central bank was communicating the projections.

Note that neither the policy rule nor the projections are optimal or consistent with the central bank’s objectives of zero output gap or inflation.^13^ The central bank is not behaving or communicating as an optimal central banker, but rather following an ad-hoc rule. Similar to projections published by real-world central banks, our experimental projections do not assume agents update their expectations in response to the communicated information. There are reasons why the central bank may still prefer to communicate these projections. The projections provide salient information that non-rational participants can base their otherwise potentially unstable expectations. The ADProj, while incorrect even if participants follow it, looks more realistic than those in the IRProj and DualProj and may be perceived as more credible.

To ensure consistency across treatments, we preselected six shock sequences (one for each session) and employed them across all treatments. The shock sequences, $r_{t}^n$ for $t=1, 2, \ldots 30$, while drawn from the same distribution with a standard deviation of 138 basis points, differed in their variability ranging from a standard deviation of 125 to 155 basis points. Varying the shock sequences across sessions allowed for a more robust analysis of expectation formation and also provided an additional dimension of exogenous variation.

### Hypotheses

The experimental design allows us to test hypotheses regarding how the aggregate economies and individual expectations evolve both with and without projections. Mainstream macroeconomic models widely assume that households and firms exhibit rational expectations about the future (Lucas 1972; Fischer 1977). Under this assumption, participants only need to rely on parameters of the model and the current shock, both of which are common knowledge, to formulate their forecasts. Figure 2 presents simulated impulse responses to a positive one-time, one-standard deviation innovation to the natural rate of interest, $r_{t}^{n}$, under alternative forecasting assumptions. All forecasting heuristics we consider are presented in Table 2. Under Model M1 Ex-Ante Rational expectations (depicted as a solid blue line), all variables increase on impact of the innovation before monotonically converging back to their steady state values as the shock to the natural rate of interest dissipates. A Dynare simulation of 10,000 draws of innovations to the natural rate of interest, $r_{t}^n$, generate standard deviations of the output gap and inflation of 101 and 30 bps, respectively, and mean absolute forecast errors of zero.

Kryvtsov and Petersen (2013) observe that aggregate expectations in an identically calibrated experiment can be well-described by an Adaptive(1) heuristic. The simulated impulse response functions of the Model M2 Adaptive(1) heuristic are depicted as red dashed lines. Compared to rational expectation (RE), aggregate forecasts of the output gap and inflation under an Adaptive(1) heuristic initially under-react to the innovation and overreact in subsequent periods. The adaptive heuristic produces a hump-shaped dynamic for both types of forecasts. Simulations show that inflation is three times more volatile than under RE with a standard deviation of 91 bps. The output gap is relatively more stable than under RE (s.d. of 85 bps), over-shoots the steady state, and becomes depressed before reverting to zero. This dampened reaction of the output gap is due to the central bank’s relatively significant interest rate reaction to deviations of inflation from its target. Overall, Adaptive(1) heuristics are associated with mean absolute forecast errors of roughly 33 (21) bps for the output gap (inflation).

Extensive survey and experimental evidence suggest that individuals do not form macroeconomic expectations rationally but instead heterogeneously weigh historical information in their forecasts (Assenza et al. 2019; Pfajfar and Santoro 2010; Pfajfar and Zakelj 2014; Coibion and Gorodnichenko 2015; Malmandier and Nagel 2016). These findings suggest a potential role for central bank communication to alleviate information frictions. While both nominal interest rate and dual projections based on the REE solution contain redundant information to a subject that fully understands the economy’s data-generating process, they may provide auxiliary assistance in forecasting the output gap and inflation for boundedly rational subjects (Simon 1959).

In our experiment, we incentivize participants to forecast accurately given their knowledge of the data-generating process and expectations of other participants’ forecasts. Commonly observed projections provide an additional focal point for subjects to coordinate their forecasts. If a subject believes that the majority of participants will utilize the central bank’s rational prediction in their forecast, her payoff-maximizing response would be to utilize the projection as her forecast. Thus, we expect participants to form more model-consistent, rational expectations in IRProj and DualProj than in NoComm. On average, this should lead to lower absolute forecast errors in the two projection treatments. In turn, we expect participants to exhibit less disagreement in their forecasts when presented with rationally-constructed projections.

The ease in effectively using the information in the projections is not the same across treatments. Subjects can effortlessly employ dual projections of the output gap and inflation as their private macroeconomic forecasts. By contrast, they must employ significant cognitive effort to infer the implied output gap and inflation projections from the communicated nominal interest rate projection. Cognitive and time limitations may lead subjects to pay relatively more attention to information that is of higher value to their payoffs and easier to process [see Simon (1959); Mazzotta and Opaluch (1995); Sims (2003); Gabaix (2014) for models of bounded rationality associated with limited processing]. Thus, we predict that rational projections of the output gap and inflation are significantly more effective at guiding aggregate expectations and the economies to the rational expectations equilibrium solution.

A subject who uses the central bank’s ADProj projection to formulate her forecasts would follow a rule given by Model M3 ADProj. That is, she would behave as if she were using the period $t-1$ inflation and output gap, as well as current innovations to formulate her period $t+1$ forecasts. If the median subjects were to use the ADProj projections, the standard deviations of the output gap (inflation) would be 84 (92) bps, hardly different from under Adaptive(1) forecasting but considerably different from Ex-Ante Rational expectations.

Adoption of the ADProj projections is a suboptimal strategy as it would lead to, on average, incorrect forecasts. Mean absolute forecast errors for the output gap (inflation) would be 18 (20) bps. Instead, the payoff-maximizing response to median forecasters using the ADProj projections would be Model M4 Best response to ADProj. A subject following M4 reacts to the relatively volatile median inflation expectations (and consequently inflation) by forming even more volatile inflation expectations. Because interest rates would rise to stabilize inflation, a payoff-maximizing strategy to median ADProj expectations would be to form more muted output gap expectations.

We simulate the case where half of the participants use the central bank’s ADProj projection while the remaining half forecast according to the ex-post rational solution (i.e. respond to aggregate expectations). The dotted green line shows the dynamics associated with this hybrid case. Compared to the fully Adaptive(1) model, expectations in this hybrid case are considerably more reactive to current innovations as a consequence of “rational” agents responding to the ADProj agents. The mixture of these two types leads to considerably greater inflation volatility on impact of the innovation and simultaneously a significant increase in the nominal interest rate and a decrease in the output gap. The standard deviations of inflation increase to 0.74, while the standard deviation of the output gap falls to 0.82. Compared to NoComm, we expect the deviations of aggregate expectations from the rational expectations equilibrium and their absolute forecast errors to be higher for inflation and lower for the output gap. We anticipate disagreement to be greater in ADProj than in NoComm based on the fact that the central bank is communicating an out-of-equilibrium projection.

Based on the above simulations and intuition from previous experimental findings, we formulate hypotheses about aggregate variables and individual expectations. In particular, we assume that aggregate expectations would follow an Adaptive(1) heuristic in the absence of central bank communication. IRProj and DualProj projections are assumed to nudge expectations in the direction of the M1 Ex-Ante Rational heuristic. In contrast, ADProj projections are assumed to encourage forecasts to adopt the M3 ADProj heuristic.

*Aggregate Hypotheses*

#### Hypothesis 1

The rational IRProj projections reduce (increase) the variability of inflation (the output gap) compared to NoComm.

#### Hypothesis 2

The rational DualProj projections reduce (increase) the variability of inflation (the output gap) compared to NoComm.

#### Hypothesis 3

 The adaptive ADProj projections increase (decrease) the variability of inflation (the output gap) compared to NoComm.

Successful communication depends on the central bank’s credibility in achieving its projections. We measure central bank credibility as the fraction of forecasts that coincide with the central bank’s explicit or implicit projected value. In our experiments, the automated central bank constructs its projections assuming that the median subjects form expectations according to either the REE or Adaptive(1) solution. The central bank’s projections will frequently be incorrect since future innovations to the shock process may not be zero (as they are, on average, predicted to be) and that subjects may use alternative heuristics to formulate their forecasts. If central bank forecasts are systematically erroneous, an optimizing agent should place less weight on the central bank projections when forming their expectations. As the projections become increasingly incorrect, we expect that the central bank will lose credibility.

*Individual Hypotheses*

#### Hypothesis 4

 The proportion of subjects forming model consistent (“rational”) expectations is larger (smaller) in DualProj and IRProj (ADProj) than in NoComm.

#### Hypothesis 5

 Projections in IRProj, DualProj, and ADProj reduce mean absolute forecast errors compared to NoComm.

#### Hypothesis 6

 Projections in IRProj, DualProj, and ADProj reduce forecast disagreement compared to NoComm.

#### Hypothesis 7

 The probability a subject utilizes the central bank’s projections decreases with the central bank’s past forecast errors.

**Fig. 4:**
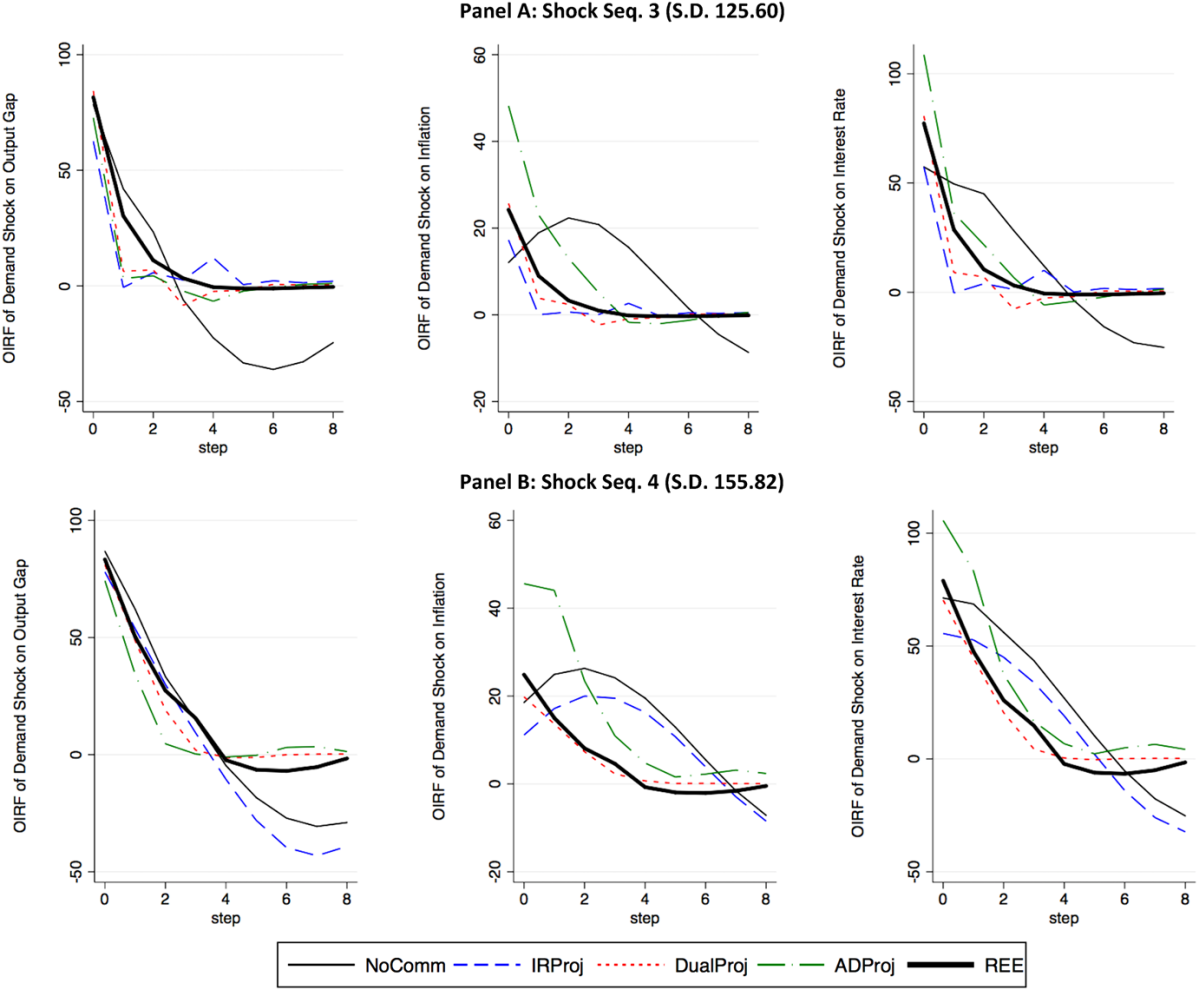
Estimated responses to a one-standard deviation innovation to the natural rate of interest. Panels A and B display estimated orthogonalized IRFs associated with the least and most volatile shock sequences in Repetition 2, respectively

**Fig. 5:**
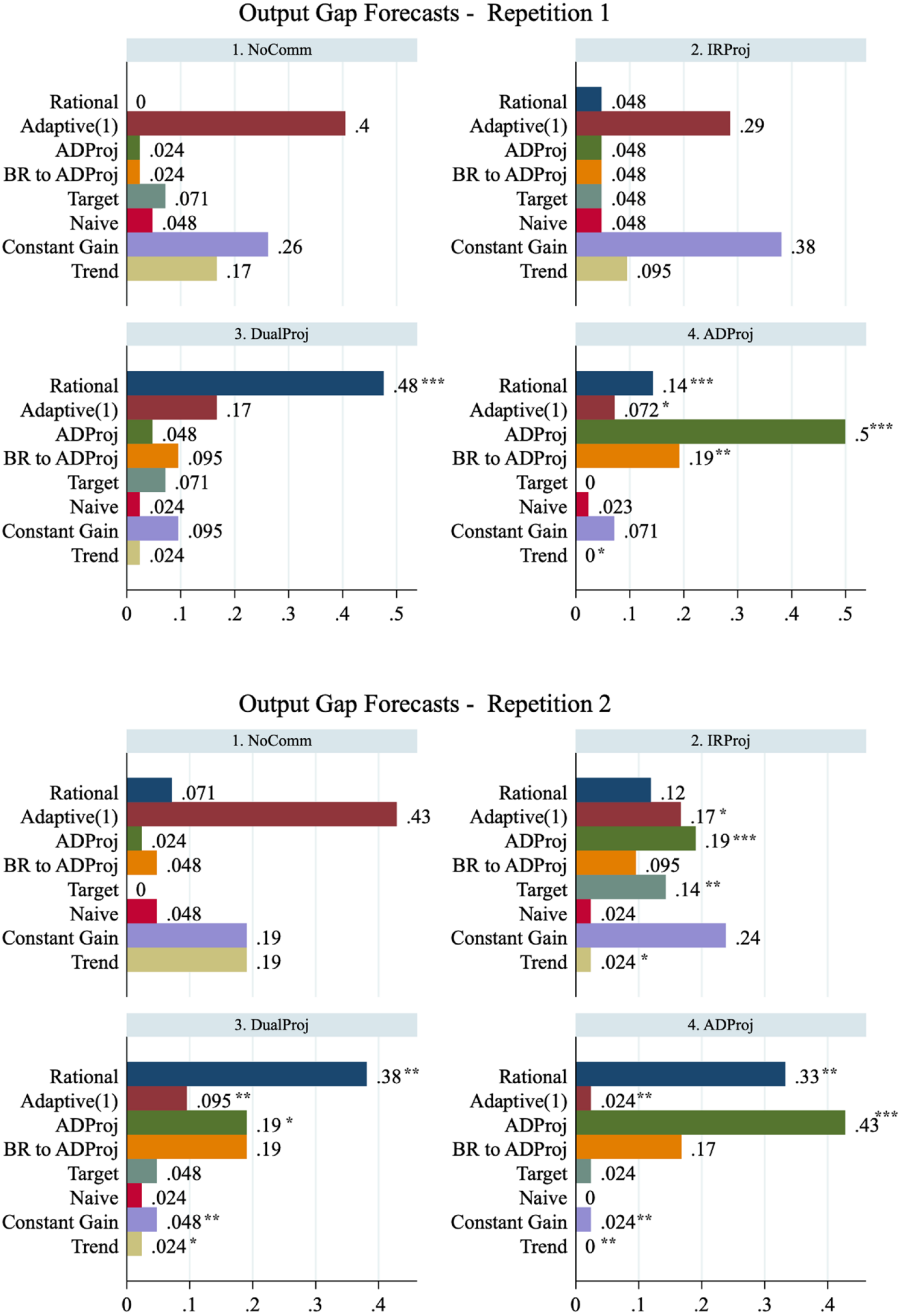
Distributions of forecasting heuristics. The figure presents the distribution of participants' output gap forecast heuristics by repetition. *p < 0:10, **p < 0:05, and ***p < 0:01 indicate that the proportion of a given type in a given treatment is significantly different from that observed in the NoComm treatment. The proportions of each type are calculated at the session level and are compared using a Wilcoxon rank-sum test (*N* = 6 observations for each treatment).* BR to ADProj* refers to Model M4, best-response to the central bank's ADProj projection

**Fig. 6:**
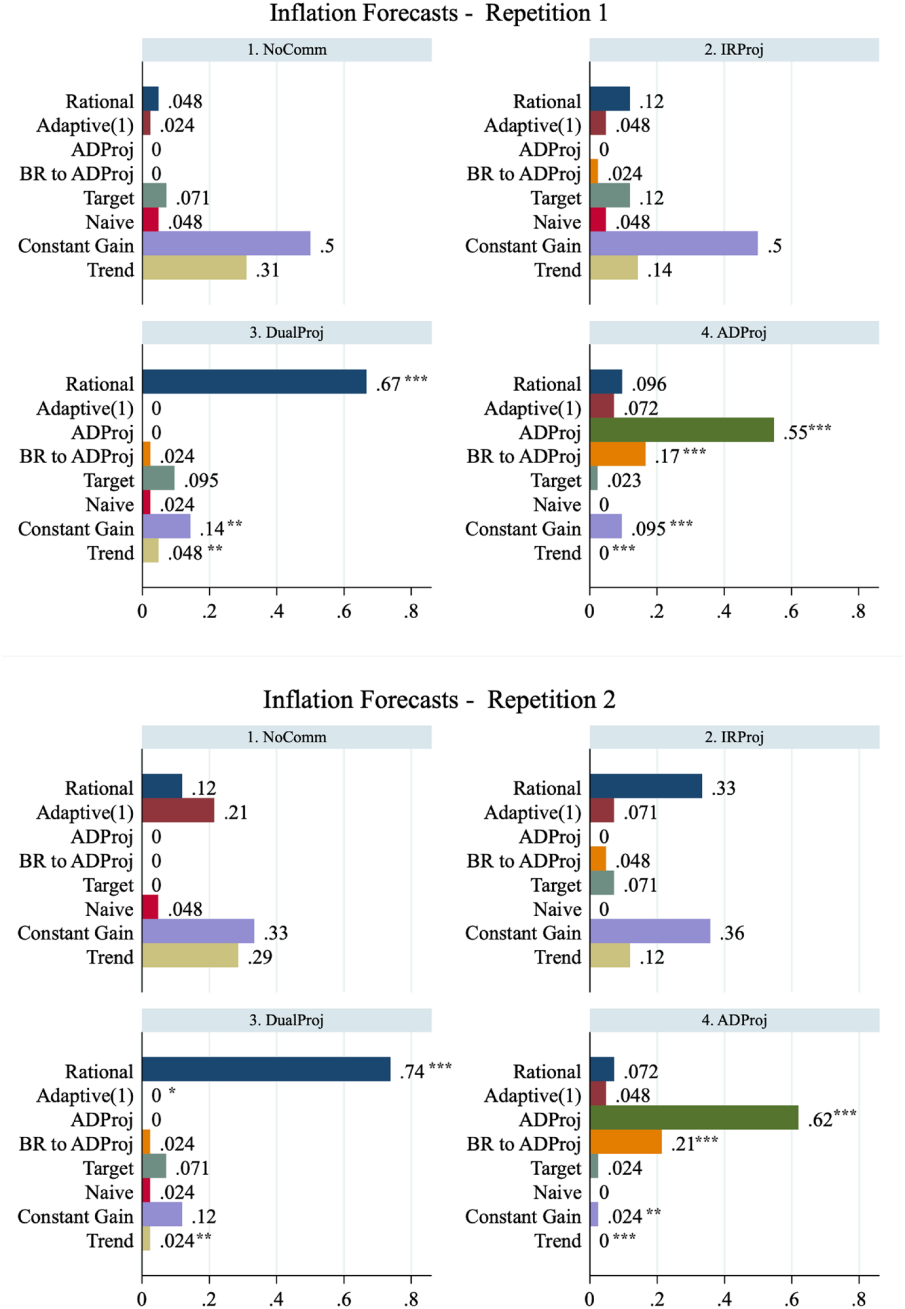
Distributions of forecasting heuristics—continued. The figure presents the distribution of participants' in ation forecast heuristics by repetition. *p < 0:10, **p < 0:05, and ***p < 0:01 indicate that the proportion of a given type in a given treatment is significantly different from that observed in the NoComm treatment. The proportions of each type are calculated at the session level and are compared using a Wilcoxon rank-sum test (*N* = 6 observations for each treatment).* BR to ADProj* refers to Model M4, best-response to the central bank's ADProj projection

**Fig. 7:**
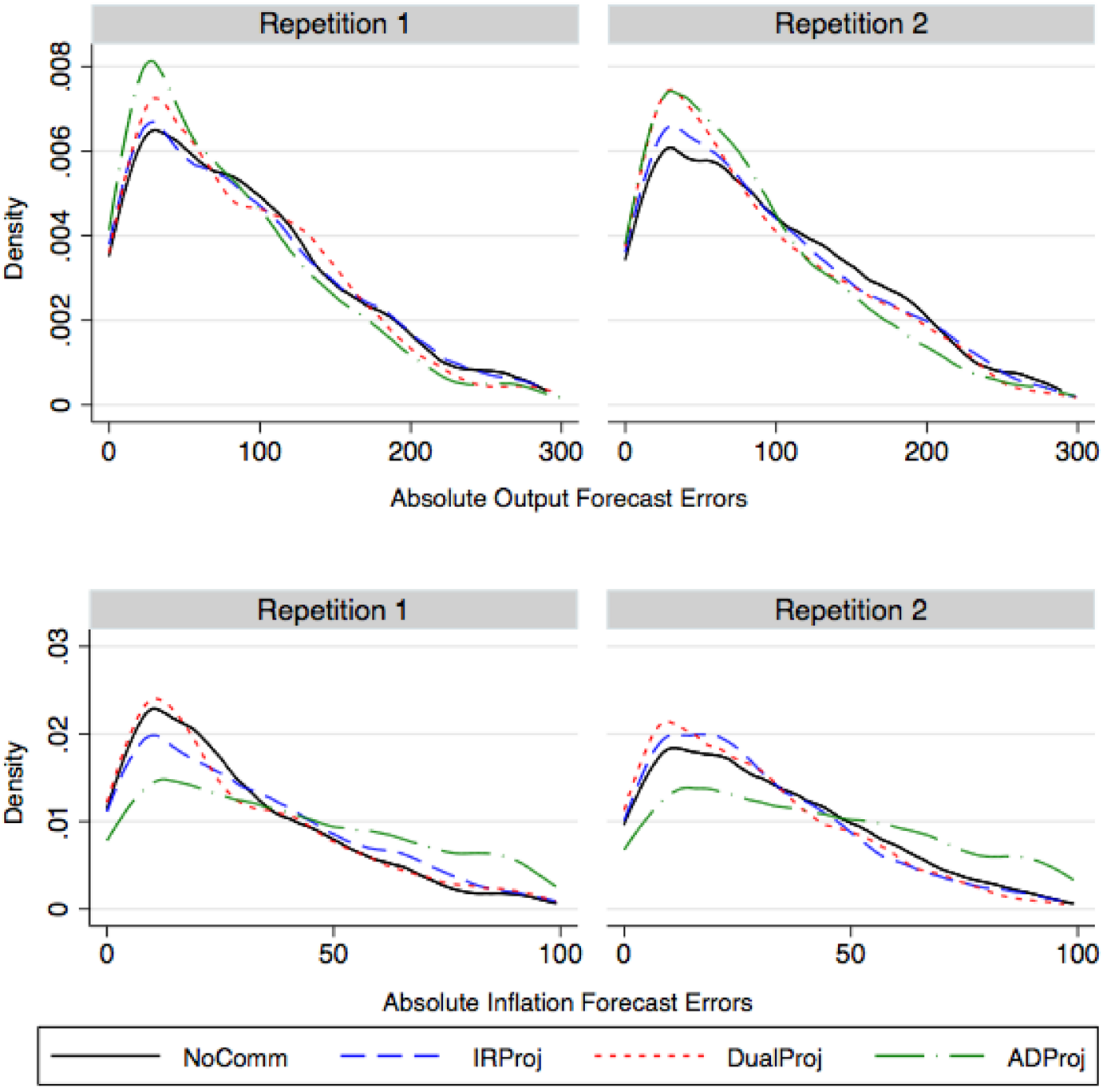
Kernel densities of absolute output and inflation forecast errors. The figure presents the kernel densities associated with individual subject's absolute forecast errors of ination and output gap across all treatments from all periods of play by repetition

**Fig. 8:**
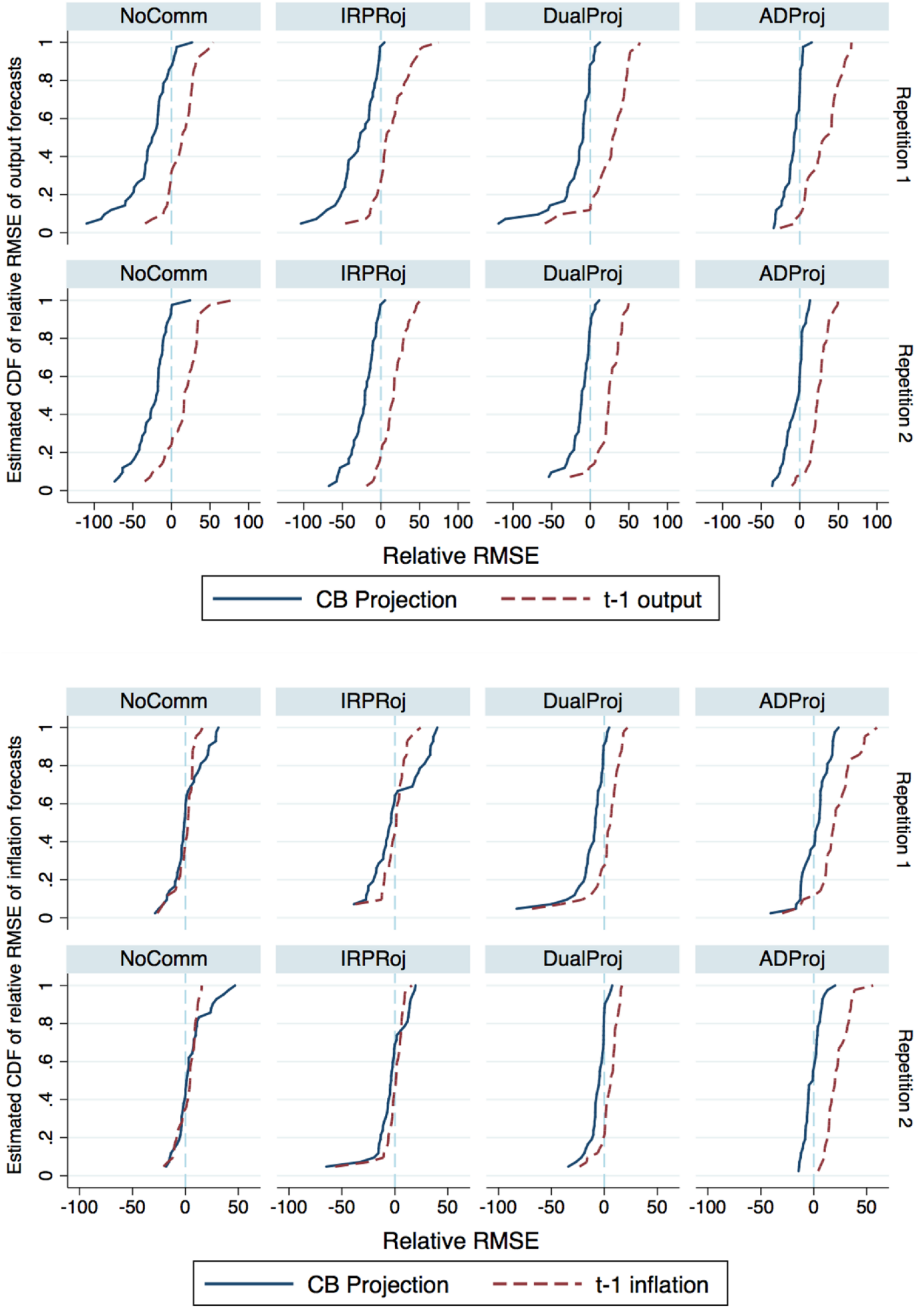
Distribution of adjustment in RMSE under counterfactual forecasting heuristics. The figure depicts the distribution of the change in the RMSE of output and ination forecasts associated with two counterfactual forecasting heuristics. For each subject in each repetition and treatment, we compute their Relative$RMSE = RMSE_{{\pi ,x}}^{{Hyp.}} - RMSE_{{\pi ,x}}^{{Actual}}$ and plot the cumulative distribution for two heuristics. The solid blue line depicts the counterfactual reduction in the RMSE associated with forecasting according to the REE solution. The dashed red line depicts the counterfactual reduction in the RMSE associated with forecasting based on the previous period's output and inflation. Negative values indicate a hypothetical improvement in forecast accuracy associated with the counterfactual heuristic.

## Experimental results

This section presents our experimental findings. We first evaluate the aggregate-level data to identify the effects of projections on economic stability and macroeconomic dynamics. We then consider how central bank (CB henceforth) projections influence subjects’ forecasting heuristics, accuracy, dispersion, and usage of the communicated information.

### Aggregate analysis

We begin by presenting representative estimated impulse response functions from our different treatments. Panels A and B of Fig. 4 displays the estimated responses of output, inflation and the nominal interest rate to a one-standard deviation innovation to the natural rate of interest in our most stable and volatile sequences in Repetition 2, respectively. The estimated impulse responses and time series from our other sessions are provided in Section C of the Online Appendix.

The thick solid black line denotes the REE solution as a reference. The estimated responses in the NoComm treatment are depicted as a thin solid black line. We observe that the output gap and inflation in the NoComm treatment deviate considerably from the REE prediction. Characteristic of an environment with Adaptive(1) aggregate expectations, inflation exhibits a distinct delayed hump-shaped pattern, and the output gap exhibits an overshooting of the steady state as the shock dissipates.

The dynamics associated with the rational IRProj treatment are presented as the thin dashed blue line, while the results from the rational DualProj treatment are presented as a thin dotted red line. In our three most stable sequences, both rational interest rate and dual projections effectively nudge expectations, and consequently, the aggregate economy to the REE solution. However, as the variability of the shocks increases in two of our three most volatile sequences, we observe that the macroeconomic dynamics revert to one consistent with adaptive expectations when the central bank communicates an interest rate projection. That is, the ability of interest rate projections to guide output and inflation expectations to the REE is limited. Rational dual projections, on the other hand, continue to work effectively even in more unpredictable environments.

The estimated impulse responses from the ADProj treatment are shown as the thin dash-dot green line. Dynamics in the ADProj treatment are consistent with our mixed model of expectations. A large fraction of agents place weight on the central bank’s adaptive dual projections of output and inflation while the remaining are ex-post rational. The output gap dynamics are slightly more stable than the REE prediction, while the inflation dynamics are significantly more volatile on impact of the innovation. Moreover, inflation exhibits a relatively monotonic transition back to the steady state (unlike under adaptive expectations).

**Table 3:** Standard deviations of the output gap and inflation normalized by the REE solution

Treatment	Repetition-1		Repetition-2	
	Std.Output gap	Std.Inflation	Std.Output gap	Std.Inflation
NoComm	1.02	1.38	1.06^**^	1.50^**^
	(0.12)	(0.62)	(0.07)	(0.41)
IRProj	0.98	1.49	0.99	1.14
	(0.13)	(0.76)	(0.15)	(0.48)
DualProj	0.96	1.06	0.97	1.04
	(0.04)	(0.20)	(0.04)	(0.12)
ADProj	0.88^**^	2.33^**^	0.88^**^	2.37^**^
	(0.05)	(0.22)	(0.03)	(0.24)
Rank-sum test	*p*-value	*p*-value	*p*-value	*p*-value
NoComm-IRProj	0.522	0.749	0.262	0.200
NoComm-DualProj	0.109	0.262	0.010	0.055
NoComm-ADProj	0.055	0.025	0.004	0.004
IRProj-ADProj	0.109	0.037	0.109	0.078
IRProj-DualProj	1.000	0.522	0.522	0.004
DualProj-ADProj	0.025	0.004	0.004	0.004

We report summary statistics (mean, standard deviation) on the variability of output and inflation, measured as the standard deviation of the variable at the session-repetition level, divided by the rational expectations equilibrium solution’s respective standard deviations. N=6 observations are computed per treatment-repetition. The top panel presents means and standard deviations of the variable of interest. Asterisks denote whether the mean result is significantly different from one using a two-sided Wilcoxon signed-rank test: $$^{*}p<0.10$$, $$^{{**}}p<0.05$$, and $$^{***}p<0.01$$. The bottom panel denotes the *p*-value results from a series of two-sided Wilcoxon rank-sum tests of identical distributions across treatments for different variables and repetitions

Summary statistics of the standard deviation of output and inflation are presented in Table 3. The standard deviations are measured at the session-repetition level and normalized by the standard deviation calculated under rational expectations for the given shock sequences.

Mean normalized standard deviations of output and inflation in the baseline NoComm treatment exceed one in both repetitions, implying the economies are, on average, more volatile than predicted by the rational expectations model. Two-sided Wilcoxon signed-rank tests are conducted to determine whether the mean results are significantly different from the REE solution, i.e. that the normalized standard deviations are equal to 1. In the first repetition of the NoComm treatment, we fail to reject the null hypothesis that the standard deviations are consistent with the REE solution. In the second repetition, the standard deviations of output and inflation in the NoComm treatment are, on average, 6% and 50% greater than the REE, respectively. This difference is significant at the 5% level. Output and inflation are not significantly different from the REE prediction at the 10% level in either the IRProj or DualProj treatments. In the ADProj treatment, output variability is significantly below the REE prediction, while inflation variability is significantly above ($p<0.05$ for both variables and repetitions).

Overall, we find mixed evidence that CB projections improve economic stability. Compared to the NoComm treatment, interest rate projections in the IRProj treatment do not significantly decrease output and inflation variability in either Repetition 1 or Repetition 2. There is considerable heterogeneity across IRProj sessions driven by differential responses of expectations to the variability of shocks. Thus, we reject Hypothesis 1 that interest rate projections affect the output gap and inflation variability.

Rational dual projections in the DualProj treatment significantly reduce output gap and inflation variability when subjects are experienced ($p=0.01$ and $p=0.055$, respectively). Adaptive dual projections in the ADProj treatment significantly stabilize output variability at the cost of significantly greater inflation variability ($p\le 0.055$ in Repetition 1, $p<0.01$ in Repetition 2). That is, we find strong support for Hypotheses 2 and 3 that dual projections influence aggregate stability, with rational DualProj reducing inflation variability and adaptive ADProj increasing inflation variability.

#### **Result 1**

*With experience, output and inflation variability in the baseline NoComm treatment is significantly greater than predicted by the REE solution. Introducing rational dual projections lowers macroeconomic variability to the REE predicted levels. Interest rate projections are not consistently effective at reducing macroeconomic variability. Adaptive dual projections reduce output variability significantly below the REE prediction while increase inflation variability significantly above it. *

### Individual-level analysis

We now evaluate how central bank projections affect individual subjects’ forecasting heuristics, forecast errors, and forecast disagreement.

#### Forecasting heuristics

 We begin by investigating how the various information treatments influence the heuristics participants use to form expectations. Table 2 lists the eight general classes of heuristics that we consider. The motivation for models M1–M4 has been discussed in Sect. 2. Model M5 Target assumes that participants simply forecast the central bank’s inflation and output gap targets each period. If subjects ignore the fact that the exogenous shock is persistent, M5 would be the REE solution. Regardless, M5 requires less cognitive effort and may not generate significant payoff losses under more stable shock sequences. Model M6 Naive assumes that participants used the previous period’s realized output gap and inflation as their forecasts. M6 is another example of a low cognitive effort strategy. In our experiment, participants have easily accessible information about past macroeconomic statistics, which has been shown in survey experiments to increase the likelihood that firms will use such information to forecast (Coibion et al. 2020). The two remaining heuristics we consider require more cognitive effort. M7 Constant Gain assumes participants will revise their past forecast upward (downward) if they under- (over-) forecasted two periods earlier. Such recursive learning models are widely used in learning-in-macroeconomics theory and applied to surveyed expectations (e.g. Evans and Honkapohja 1999; Malmandier and Nagel 2016). Finally, M8 Trend Chasing assumes participants extrapolate recent trends in output and inflation. Trend chasing heuristics are widely observed in learning-to-forecast and financial market experiments (Pfajfar and Zakelj 2014, 2016; Bao et al. 2017).

Similar to Pfajfar and Zakelj (2014), we classify a participant as ‘using’ the model that produces the lowest absolute mean-squared error among all competing models. To be clear, we are not evaluating the fit of any one model but identifying which of the eight heuristics best describes each participant’s forecasting behaviour. A range of parameters $\gamma , \tau \in [0.1,1.5]$ with 0.1 increments are used for M7 Constant Gain and M8 Trend-Chasing models. The proportion of participants that are best described by each type is presented in Figs. 5 and 6 for output gap and inflation forecasts, respectively. We compute, for each forecasted variable, the proportion of each type at the session-level and conduct a series of two-sided Wilcoxon rank-sum tests of identical distributions across treatments for different variables and repetitions ($N=6$ per treatment). Statistical significant differences in the proportion of a type are indicated with asterisks.

We begin by describing the heuristics observed in the NoComm treatment. Consistent with Kryvtsov and Petersen (2013), we find minimal evidence of M1 Ex-Ante Rational expectations. Very few subjects base their forecasts on the central bank’s explicitly communicated targets. Rather, we observe that the vast majority of participants rely on heuristics that incorporate historical information. For output gap forecasts, the most frequently observed heuristic is the M4 Adaptive(1) model. For inflation forecasts, the majority of subjects use the M7 Constant Gain or M8 Trend-Chasing heuristics. Finally, the simple M3 Naive model and M5 Target Model are used by a small fraction of subjects, indicating that most participants correctly believe that the economy will change over time.

The IRProj interest rate projection does not have a significant effect on inexperienced forecasters’ expectation formation. The share of participants following M8 Trend-Chasing heuristics decreases by roughly one-half for both variables, but the effect is not statistically significant at the session-level. However, with experience, IRProj participants significantly decrease their usage of M2 Adaptive(1) and increase their usage of the M4 ADProj and M5 Target and to forecast the output gap. The projection has no significant effect on the heuristics participants use to forecast inflation. We fail to reject the null hypothesis that the proportion of Ex-Ante Rational types is identical in the NoComm and IRProj treatments ($p>0.13$ for both variables in both repetitions). Thus, we find limited evidence to support Hypothesis 4 that IRProj increases the proportion of Ex-Ante Rational types.

In DualProj rational projections of the output gap and inflation substantially increase the prevalence of Ex-Ante Rational forecasting. At the same time, DualProj reduces the prevalence of many backward-looking heuristics (M2, M6, M7, M8). This shift in the distribution of heuristics toward more rationality persists as participants become experienced. We reject the null hypothesis that the proportion of Ex-Ante Rational types is identical in the NoComm and DualProj treatments ($p<0.012$ for both variables in both repetitions). Thus, we find evidence to support Hypothesis 4 that DualProj increases the proportion of Ex-Ante Rational types.

Finally, in ADProj, the modal heuristic is the M3 ADProj. We also observe a sizeable proportion of participants using the payoff-maximizing response to M4 ADProj. Not surprising, both of these heuristics are significantly more represented than in the NoComm treatment. Their emergence comes at a significant reduction in the prevalence of Constant Gain and Trend Chasing heuristics. It is also worth noting that the share of output gap forecasts classified as rational also increases in both repetitions. This observation is, perhaps, not surprising given that the ADProj and Ex-Ante Rational output gap forecasts are very similar in magnitude. We fail to reject the null hypothesis that the proportion of Ex-Ante Rational types is identical in the NoComm and ADProj treatments for inflation forecasts ($p>0.35$ in both repetitions) but do reject the null hypothesis for output gap forecast ($p<0.012$). Thus, we find mixed evidence to reject Hypothesis 4 that ADProj decreases the proportion of Ex-Ante Rational types.

##### **Result 2**

*The proportion of Ex-Ante Rational participants increases significantly in the DualProj treatment but not significantly in the IRProj. ADProj does not significantly decrease the proportion of Ex-Ante Rational participants.*

#### Forecast errors 

 Central bank projections are meant, among other things, to help forecasters better anticipate the future. Thus, one measure of the success of a CB’s projection is its ability to reduce forecast errors. The distributions of subjects’ absolute forecast error (the absolute difference between their forecasts and the realized outcomes) are presented in Fig. 7. We find that, for experienced participants in Repetition 2, all three types of projections skew the distribution of absolute output gap forecast errors down compared to the NoComm treatment. By contrast, the distribution of absolute inflation forecast errors is only noticeably skewed downward in the DualProj treatment, while the ADProj treatment is associated with larger forecast errors compared to NoComm.

**Table 4:** Effects of central bank projections on absolute forecast errors and disagreement—treatment effects I

	ln(Absolute forecast errors)				ln(SD of forecasts)			
	Output gap		Inflation		Output gap		Inflation	
	Rep. 1	Rep. 2	Rep. 1	Rep. 2	Rep.1	Rep. 2	Rep. 1	Rep. 2
NoComm	82.5	74	29.2	29.5	196.99	175.15	19.31	19.58
	(18.93)	(17.79)	(5.38)	(7.29)	(375.46)	(280.30)	(3.27)	(3.03)
IRProj	99.17	74.5	63.17	29	55.65	49.85	35.06	22.28
	(52.91)	(12.11)	(79.10)	(9.49)	(29.89)	(8.05)	(21.85)	(9.56)
DualProj	79.33	79.83	26.67	26	50.37	47.70	29.75	22.36
	(23.70)	(19.40)	(15.63)	(5.06)	(24.85)	(32.90)	(24.41)	(15.07)
ADProj	64.5	67.83	48.83	45.5	32.91	30.07	26.40	21.91
	(15.93)	(14.12)	(16.62)	(13.19)	(4.05)	(3.95)	(6.22)	(5.30)
*Rank-sum test*								
NoComm-IRProj	0.57	0.81	0.42	0.52	0.87	0.11	0.02	0.75
NoComm-DualProj	0.20	0.26	0.93	0.33	0.52	0.05	0.63	0.26
NoComm-ADProj	0.17	0.15	0.00	0.00	0.05	0.00	0.04	0.20
IRProj-DualProj	0.30	0.20	0.34	0.75	0.52	0.08	0.26	0.52
IRProj-ADProj	0.11	0.13	0.02	0.00	0.02	0.00	0.75	0.63
DualProj-ADProj	0.04	0.57	0.00	0.00	0.15	0.08	0.75	0.26

(I) The table presents summary statistics (mean, standard deviation) of median forecast errors and median disagreement both calculated at the treatment-session-repetition level. The bottom panel denotes the *p*-value results from a series of two-sided Wilcoxon rank-sum tests of identical distributions across treatments for different variables and repetitions

We provide summary statistics on median absolute forecast errors in each session in the first four columns of Table 4. We focus on median forecast errors as they are less influenced by extreme outlier forecasts. In the NoComm treatment, median output gap forecast errors decline from 82.5 in Repetition 1 to 74 bps in Repetition 2 as participants become more experienced. Inflation forecast errors hardly change and are roughly 29 basis points across the two repetitions.

Compared to NoComm, forecast errors increase on average in IRProj when participants are inexperienced for the output gap (inflation) to 99 (63) bps. With experience, IRProj errors decrease and the differences across treatments are negligible (less than a basis point). For both inexperienced and experienced participants, the differences are not statistically significant (Wilcoxon rank-sum test, $N=6$ per treatment, $p>0.42$ in all pairwise comparisons).

Likewise, DualProj does not significantly influence forecast accuracy irrespective of participants’ experience. The differences in forecast errors across NoComm and DualProj are less than 5 basis points in magnitude and not statistically significant ($p\ge 0.20$ in all cases).

ADProj projections do, however, improve output gap forecast accuracy while worsening inflation forecast accuracy. Median forecast errors for the output gap fall to between 64 and 67 bps but this decrease relative to NoComm is not statistically significant. The improvement in output gap forecast accuracy is mainly coming from the increased stability in the output gap. Inflation forecast errors nearly double to between 45 and 48 bps. The differences in inflation forecast accuracy between NoComm and ADProj are highly significant for both inexperienced and experienced participants ($p<0.01$).

Together, these results lead us to reject Hypothesis 5 that projections do not improve forecast accuracy.

##### **Result 3**

*Projections do not significantly improve forecast accuracy, and in the case of ADProj, significantly worsen inflation forecast accuracy.*

#### Forecast disagreement

 Next, we evaluate whether the central bank projections provide a sufficient focal piece of information for subjects to coordinate their forecasts. We quantify the degree of disagreement among subjects by calculating the standard deviation of forecasts each period across subjects in a single session. Summary statistics by variable and repetition are presented in columns (5)–(8) of Table 4.

NoComm disagreement about future output gaps falls from a mean of 197 to 175 bps as participants become more experienced. There is a negligible change in inflation disagreement, with a standard deviation of roughly 20 bps in both repetitions.

On average, all the projections reduce output disagreement and increase inflation disagreement. Most notably, inflation disagreement is 85% higher in IRProj than NoComm when participants are inexperienced. This effect is statistically significant at the 5% level. By contrast, DualProj projections significantly reduce output gap disagreement when participants are experienced ($p=0.05$) but do not have a consistent effect on inflation forecasts. Finally, ADProj significantly reduces output gap disagreement when participants are experienced ($p<0.01$) and increases inexperienced inflation disagreement ($p<0.05$). Thus, we obtain mixed support for Hypothesis 6.

##### **Result 4**

*Projections reduce output gap disagreement and increase inflation disagreement.*

#### Central bank accuracy and credibility

 Across the projection treatments, the central bank’s mean absolute forecast errors for the output gap range from 77 to 79 basis points, with no significant differences across any treatment-repetition comparisons ($p>0.50$ in all pairwise Wilcoxon rank-sum tests). Central bank mean absolute inflation forecast errors are the lowest in the DualProj at 24 basis points, followed by 33 basis points in the IRProj, and 56 basis points in the ADProj treatments. The difference between the DualProj and ADProj is statistically significant at the 1% level, while the differences between the IRProj and ADProj are significant at the 5% level.

Credibility is an important concern for central banks in their decision to publish their internal forecasts. We describe a central bank’s projections as credible if subjects utilize it as their private forecast. Our variables of interest are $UtilizedCBxForecast_{t}$ and

$UtilizedCB \pi Forecast_{t}$ which take the value of 1 if a subject’s period *t* forecast about $t+1$ was less than five basis points from the central bank’s projection and zero otherwise.^14^ The percentage of utilization is presented at the bottom of Table 5. Interest rate projections appear to be ineffectively utilized, with only 7% (13%) of output gap (inflation) forecasts in line with the projection. By contrast, the utilization of rational and adaptive dual projections is considerably higher. Output gap (inflation) projections in DualProj are utilized in 25% (38%) of the submitted forecasts. Likewise, ADProj projections are utilized in 28% (45%) of the output gap (inflation) forecasts. Differences in utilization between the DualProj and ADProj treatments are only statistically significant for output forecasts ($N=6$, two-sided Wilcoxon rank-sum test, $p<0.05$ for both repetitions). IRProj is significantly less utilized than DualProj or ADProj ($p<0.01$ for each treatment-repetition-variable test).

**Table 5:** Credibility and disagreement in Central Bank projections of output and inflation—by treatment

	Dep.Var: Prob(Utilized CB Forecast=1)						ln(SD of Forecasts)					
	IRProj		DualProj		ADProj		IRProj		DualProj		ADProj	
	$E_{i,t} x_{t+1}$	$E_{i,t} \pi _{t+1}$	$E_{i,t} x_{t+1}$	$E_{i,t} \pi _{t+1}$	$E_{i,t} x_{t+1}$	$E_{i,t} \pi _{t+1}$	$E_{t}x_{t+1}$	$E_{t}\pi _{t+1}$	$E_{t}x_{t+1}$	$E_{t}\pi _{t+1}$	$E_{t}x_{t+1}$	$E_{t}\pi _{t+1}$
	(1)	(2)	(3)	(4)	(5)	(6)	(7)	(8)	(9)	(10)	(11)	(12)
$|FE^{cb} x_{t-1}|$	$-$0.004$^*$		$-$0.001		$-$0.002		0.001		0.004$^*$		0.000	
	(0.00)		(0.00)		(0.00)		(0.00)		(0.00)		(0.00)	
$|FE^{cb} x_{t-1}|^2$	0.000		0.000		0.000		0.000$^*$		$-$0.000		0.000	
	(0.00)		(0.00)		(0.00)		(0.00)		(0.00)		(0.00)	
$$Utilized CB x Forecast_{t-1}$$	0.093		0.375$$^{***}$$		0.220$$^{**}$$							
	(0.16)		(0.11)		(0.09)							
$$|FE x_{i,t-1}|$$	0.001		$$-$$0.002$$^{***}$$		$$-$$0.001							
	(0.00)		(0.00)		(0.00)							
$$|FE x_{i,t-1}|\times Utilized CB x Forecast_{t-2}$$	0.001		0.002$$^*$$		0.002$$^{***}$$							
	(0.00)		(0.00)		(0.00)							
SD $$r^{n}_{t}$$	$$-$$0.011$$^{***}$$	$$-$$0.004	0.001	0.003	$$-$$0.003	$$-$$0.002	0.013$$^{***}$$	0.010$$^{***}$$	0.003	$$-$$0.007	$$-$$0.001	0.001
	(0.00)	(0.00)	(0.01)	(0.01)	(0.01)	(0.01)	(0.00)	(0.00)	(0.00)	(0.00)	(0.00)	(0.00)
Experienced	0.147$$^*$$	0.032	0.023	0.068	0.100	$$-$$0.025	$$-$$0.079	$$-$$0.355$$^{***}$$	$$-$$0.115	$$-$$0.179$$^*$$	$$-$$0.122$$^*$$	$$-$$0.169$$^{**}$$
	(0.08)	(0.09)	(0.18)	(0.18)	(0.16)	(0.15)	(0.05)	(0.08)	(0.09)	(0.10)	(0.07)	(0.07)
$$|FE^{cb} \pi _{t-1}|$$		$$-$$0.012$$^{***}$$		$$-$$0.004		$$-$$0.008$$^{***}$$		0.005$$^*$$		0.007		0.006$$^{**}$$
		(0.00)		(0.00)		(0.00)		(0.00)		(0.01)		(0.00)
$$|FE^{cb} \pi _{t-1}|^2$$		$$-$$0.000		$$-$$0.000		0.000$$^{***}$$		$$-$$0.000		$$-$$0.000		$$-$$0.000
		(0.00)		(0.00)		(0.00)		(0.00)		(0.00)		(0.00)
$$Utilized CB \pi Forecast_{t-1}$$		0.274$$^{***}$$		0.450$$^{***}$$		0.363$$^{***}$$						
		(0.09)		(0.09)		(0.07)						
$$|FE \pi _{i,t-1}|$$		0.000		$$-$$0.004$$^{***}$$		$$-$$0.003$$^{***}$$						
		(0.00)		(0.00)		(0.00)						
$$|FE \pi _{i,t-1}|\times Utilized CB \pi Forecast_{t-2}$$		0.002		0.006$$^{**}$$		0.001						
		(0.00)		(0.00)		(0.00)						
$$\alpha$$	$$-$$0.004	$$-$$0.312	$$-$$0.905	$$-$$0.723	$$-$$0.063	0.152	2.023$$^{***}$$	1.889$$^{***}$$	3.161$$^{***}$$	3.950$$^{***}$$	3.522$$^{***}$$	2.936$$^{***}$$
	(0.48)	(0.54)	(1.02)	(1.02)	(0.88)	(0.82)	(0.31)	(0.51)	(0.57)	(0.71)	(0.38)	(0.43)
% Observations where Utilized CB Forecast=1	0.07	0.13	0.25	0.38		0.22	0.42					
Average CB Forecast Error (basis points)	77	33	79	24		78	56					
$N$	2346	2346	2342	2342	2277	2277	336	336	336	336	328	328
$\chi ^2$	36.98	47.79	30.14	62.08	25.25	55.40	75.71	56.70	11.48	5.204	26.82	20.96
*Random effects*												
subject	$\checkmark$	$\checkmark$	$\checkmark$	$\checkmark$	$\checkmark$	$\checkmark$						
session					$\checkmark$	$\checkmark$		$\checkmark$	$\checkmark$	$\checkmark$	$\checkmark$	

This table presents results from a series of probit regressions to evaluate the drivers of credibility

Random effects at the subject level are included in columns (1)–(6) while session level are employed in columns (7)–(12). Robust standard errors are employed. *$p<0.10$, **$p<0.05$, and ***$p<0.01$. $Utilized CB x Forecast_{t-1}$ and $Utilized CB \pi Forecast_{t-1}$ are dummy variables that take the value of one if a subject’s output and inflation forecast in period $t-1$ about period *t*, respectively, were less than five basis points away from the central bank’s projected forecast. $|FE^{cb} x_{t-1}|$ and $|FE^{cb} \pi _{t-1}|$ denote the absolute forecast errors the central bank made in period $t-2$ about period $t-1$ output and inflation, respectively. $|FE x_{i,t-1}|$ and $|FE \pi _{i,t-1}|$ denote subject *i*’s forecast errors formed in period $t-2$ about period $t-1$ output and inflation, respectively. By comparison, NoComm forecasts are within 5 basis points of the REE solution for 6% of output forecasts and 11% of inflation forecasts

We estimate a series of panel probit models to understand how the likelihood a subject utilizes the central bank’s projections evolves. Our primary explanatory variables are the central bank’s absolute forecast errors about period $t-1$ output, $|FE^{cb} x_{t-1}| = |E^{cb}_{t-2}x_{t-1}-x_{t-1}|$ and $t-1$ inflation, $|FE^{cb} \pi _{t-1}| =|E^{cb}_{t-2}\pi _{t-1}-\pi _{t-1}|$. We additionally control for subjects’ previous utilization of the central bank’s forecast in period $t-2$ and subjects’ own absolute forecast errors $|FE x_{i,t-1}|$ and $|FE \pi _{i,t-1}|$, and interactions of these two variables. We pool together data from both repetitions, as the differences across repetitions are unnoteworthy. Subject random effects are included and robust standard errors are employed. Treatment-specific results are presented in the first six columns of Table 5.

We find mixed support for Hypothesis 7 that central bank credibility decreases with larger forecast errors. In the IRProj treatment, the probability that a subject uses the central bank’s interest rate projection for either forecast decreases significantly when the central bank makes larger forecast errors. Likewise, in the ADProj treatment, larger central bank forecast errors about inflation significantly reduce subjects’ utilization of its inflation projections. By contrast, credibility in the DualProj treatment is not significantly affected by central bank forecast errors.

##### **Result 5**

*Credibility decreases significantly when the central bank makes larger forecast errors while communicating either an interest rate projection or an adaptive dual projection, but not when it is communicating rational dual projections.*

Last, we estimate the effects of past central bank forecast errors on the dispersion in subjects’ forecasts using a series of probit regressions with session random effects. The results are presented in the final six columns of Table 5. Larger past errors by the central bank lead to increased disagreement about future inflation in all three projection treatments, with the effects statistically significant and large in IRProj and ADProj. Volatility in the environment measured by the standard deviation of the shock process, SD $r_t^n$, only appears to increase disagreement in IRProj. In DualProj and ADProj, the projections appear to work well as a coordination device even as the economy becomes more unpredictable.

## Discussion

Central banks are increasingly incorporating household heterogeneity into their forecasting models to better capture realistic aggregate dynamics. While a combination of rational and backward-looking expectations are well-supported by survey and experimental data, our findings suggest that central banks interested in maintaining inflation stability in the presence of demand shocks should strategically communicate projections based solely on rational expectations. Rationally-constructed projections would encourage naïve agents to form more stable inflation expectations and reduce inflation variability.

Additionally, central bank communication must be easy to understand. Rational projections of output and inflation reduce subjects’ backward-looking forecasting heuristics and refocus their expectations on current fundamentals. Such announcements lead to reduced heterogeneity in forecasts and somewhat smaller forecast errors. By contrast, projections of nominal interest rates are inconsistently effective at coordinating expectations and improving forecast accuracy, especially when it comes to inflation forecasts. Our experimental results contribute to a growing literature in central bank communication has shown that relevant and straightforward to understand communications are more effective at managing expectations. For instance, in a recent Bank of England survey experiment, participants were much more likely to comprehend central bank communication and convey trust in the BoE when presented with simple, relatable information (Bholat et al. 2019).

The fact that nominal interest rate projections are more challenging for subjects to utilize than dual macroeconomic projections can be understood through the lens of models of rational inattention. Such models assume that agents, with a limited amount of attention, continuously receive imperfect information in the form of noisy signals about the state of the economy, but must optimally choose which information to pay close attention to and which information to ignore (Sims 2003; Mackowiak and Wiederholt 2009).^15^

Rational inattention models predict that the optimal allocation of limited attention to information is decreasing in the marginal cost of processing that information. In DualProj and ADProj, subjects can employ the explicitly communicated output and inflation projection with relative ease. In contrast, IRProj interest rate projections require more time and cognitive effort to translate into output and inflation projections. Indeed, we find that subjects are roughly three times more likely to effectively utilize inflation and output gap projections than interest rate projections when forming their expectations.

Rational inattention models also assume that agents equate the marginal cost of paying attention to projections to the marginal benefit of using such projections. That is, subjects would optimally pay less attention to information that is unlikely to adequately compensate them for the effort of processing such information.

To evaluate this prediction, we compute a set of counterfactual payoffs where we assume that the subject either uses the central bank’s projection or period $t-1$ output and inflation as its forecast. We select period $t-1$ output and inflation as counterfactuals because historical information appears to play a dominant role in subjects’ forecasts.^16^ For each subject, we compute the root mean squared errors (RMSE) the subject would have incurred had they forecasted under either of these alternative heuristics holding constant other subjects’ forecasting behavior. We subtract from the counterfactual RMSE their actual RMSE to compute a relative RMSE. A negative RMSE implies that a subject would have improved her forecasting performance by adopting an alternative forecasting heuristic, and vice versa. Figure 8 plots the cumulative distribution of subjects’ relative RMSEs for each of the two counterfactual forecasting heuristics by treatment and repetition. We include counterfactual cumulative distributions for the NoComm treatment assuming they either forecasted according to the REE solution or naïvely.

When forecasting output, the vast majority of subjects in all treatments would have improved their payoffs by forecasting according to the central bank’s projection. Our results suggest that while most subjects are not optimally utilizing the central bank projections, the irrational inattention observed in DualProj and ADProj is rather low. Moreover, subjects rationally avoided using purely naïve strategies that would have decreased their accuracy.

The results for inflation forecasts in the NoComm and IRProj treatments are considerably different. The majority of experienced NoComm subjects would have made larger forecast errors by individually employing the REE solution as their forecast. That is, a strategy that would have had subjects respond more to the innovations would have led them to over-react relative to their fellow forecasters and generate larger forecast errors. A similar pattern emerges for 25% of experienced IRProj subjects. Given that most IRProj subjects in sessions with greater shock volatility were not actively employing the *implied * inflation projection as their forecast, responding to the nominal interest rate projection would have led to more substantial forecast errors. Put another way, these IRProj subjects *rationally* ignored the interest rate projection.

Many central banks are now entertaining alternative policy regimes as their policy rates creep toward their effective lower bounds. Price-level and nominal GDP targeting are two possible candidates that can significantly improve macroeconomic stability. These policies, however, demand a high level of rationality to be implemented effectively. Our results suggest that rationally-constructed projections can nudge individuals to forecast rationally and may serve as a critical communication component in a successful regime shift.

## Electronic supplementary material

Below is the link to the electronic supplementary material.

Supplementary material 1 (pdf 3954 KB)

## Footnotes

1 Ferrero and Secchi (2010) discuss the widespread strategies of central banks to employ rational expectations into their core macroeconomic DSGE models. As they note, there is an awareness that the general public does not form rational expectations, and efforts need to be made to bring this realism into projection models. To date, the Bank of Canada, Bank of Israel, Norges Bank, and Riksbank’s main projection models are built around the assumption of rational expectations. The Bank of England’s COMPASS, the Reserve Bank of New Zealand’s NZSIM, and the ECB’s New Multi-Country Model incorporate extensions allowing for adaptive expectations (Kravik and Yasin 2019; Dorich et al. 2013; Constncio 2017; Monti 2018; Kamber et al. 2015; Burgess et al. 2013).

2 The LtFE methodology originates with Marimon and Sunder (1993), who study price forecasting in an overlapping-generations experimental economy. Experiments studying inflation and output gap expectations in New Keynesian reduced form economies have been developed to study expectation formation and equilibria selection (Adam 2007); the effects of different monetary policy rules on expectation formation (Pfajfar and Zakelj 2014, 2016; Assenza et al. 2019; Hommes et al. 2019a); expectation formation at the zero lower bound (Arifovic and Petersen 2017; Hommes et al. 2019b). Backward-looking, inattentive forecasting behavior frequently observed in laboratory experiments is also widely found in household and professional forecasts (Malmendier and Nagel 2016; Andrade and Le Bihan 2013; Coibion and Gorodnichenko 2015; Cornand and Hubert 2020).

3 See Duffy (2012) for a highly comprehensive survey of macroeconomic experiments, Cornand et al. (2014) for a survey of experiments on central banking, and Amano et al. (2014) for a discussion of how laboratory experiments can help inform monetary policy.

4 See Walsh (2010) for detailed assumptions and derivations in a model with rational expectations. We preferred to implement a linearized version of the homogeneous expectations New Keynesian model to simplify the environment for subjects. For a nonlinear implementation, see Hommes et al. (2019b) . For a heterogeneous version of the model, see Mauersberger (2017) and Kryvtsov and Petersen (2020). While this system could be simplified and written as a function of just one- and two-period ahead inflation forecasts (Rholes and Petersen 2020), we preferred to capture the fact that people must form expectations about multiple variables when making economic and financial decisions.

5 The natural rate of interest is the equilibrium real rate of interest required to keep aggregate demand equal to the natural rate of output at all times.

6 We differ from Kryvtsov and Petersen (2013) who implement a policy rule that responds to deviations of past expected inflation and expected output gaps from the central bank’s target policy.

7 See Arifovic and Petersen (2017), Hommes et al. (2019b), and Ahrens et al. (2016) for analysis of expectations in an environment with a binding lower bound on interest rates.

8 Instructions can be found in Section A of the Online Appendix. Mirdamadi and Petersen (2018) demonstrate that precise quantitative information about the data-generating process reduces inflation forecast dispersion and forecast errors.

9 As is common in macroeconomics experiments, we provided contextual framing in the instructions and environment to encourages participants to bring their own experiences and understanding into the lab and to weaken the experimenter’s control over the environment (Alekseev et al. 2017). Given our interest in understanding how *central bank* communication influences expectations, it seemed appropriate to employ more realistic language.

10 In each treatment, the median proportion of periods that a participant is the median forecaster ranges between 17 and 20%. Likewise, the mean proportion ranges from 17 to 21%. The proportion is slightly higher than 14.3% (1/7), the predicted number of rounds under random assignment to the median. This suggests that some participants are more likely to be “middle of the road” in their forecasts, and have more influence on the aggregate economy.

11 See Rholes and Petersen (2020) for experimental evidence on the effects of communicating confidence intervals around inflation projections.

12 We report the results of additional treatments involving individual rational output gap or inflation projections in Mokhtarzadeh and Petersen (2015).

13 An interesting alternative projection would involve the central bank communicating a projection based on the optimal policy of $E^{CB}_{t}[\pi _{t+1}]=0$ and $E^{CB}_{t}[x_{t+1}]=-\frac{1}{\sigma }r_{t}^n$. If private agents use these forecasts as their own, then $\pi _{t}=x_{t}=0$ at all dates. We leave this policy experiment to further research.

14 We are implicitly assuming that subjects fully comprehend how to utilize the central bank’s interest rate projection to formulate their output and inflation forecasts.

15 See Section C of the Online Appendix for a discussion of alternative models of recursive learning and sticky information to rationalize our results.

16 In the DualProj and ADProj treatments, the marginal cost associated with employing the central bank’s projection or period $t-1$ output and inflation and output as one’s forecast is comparable. Subjects simply have to move their mouse over either value and input those values into the experimental interface. In the IRProj treatment, computing the implied forecast for output and inflation from the interest rate projection is considerably more challenging than using historical values, and would arguably exhibit a larger marginal cost for the subject.

## References

[CR1] Adam K (2007). Experimental evidence on the persistence of output and inflation. Economic Journal.

[CR2] Ahrens, S., Lustenhouwer, J., & M. Tettamanzi. (2016). The stabilizing role of forward guidance: A macro experiment, BERG Working Paper Series 137, Bamberg University, Bamberg Economic Research Group.

[CR3] Alekseev A, Charness G, Gneezy U (2017). Experimental methods: When and why contextual cues are important. Journal of Economic Behavior and Organization.

[CR4] Amano R, Kryvtstov O, Petersen L (2014). Recent developments in experimental macroeconomics.

[CR5] Andrade P, Le Bihan H (2013). Journal of Monetary Economics.

[CR6] Armantier O, Bruine de Bruin W, Topa G, van der Klaauw W, Zafar B (2015). Inflation expectations and behavior: Do survey respondents act on their beliefs?. International Economic Review.

[CR7] Assenza, T., Heemeijer, P., Hommes, C. H., & Massaro, D. (2019). Managing self-organization of expectations through monetary policy: A macro experiment. Journal of Monetary Economics.

[CR8] Arifovic J, Petersen L (2017). Stabilizing expectations at the zero lower bound: Experimental evidence. Journal of Economic Dynamics and Control.

[CR9] Bao T, Hommes C, Makarewicz T (2017). Bubble formation and (In) efficient markets in learning-to-forecast and optimise experiments. The Economic Journal.

[CR10] Bholat D, Broughton N, Ter Meer J, Walczak E (2019). Enhancing central bank communications using simple and relatable information. Journal of Monetary Economics.

[CR11] Brubakk, L., ter Ellen, S., & Xu, H. (2017). Forward guidance through interest rate projections: Does it work? Norges Bank Working Paper 6/2017.

[CR12] Burgess, S., Fernandez-Corugedo, E., Groth, C., Harrison, R., Monti, F., Theodoridis, K., & Waldron, M. (2013). The Bank of England’s forecasting platform: COMPASS, MAPS, EASE and the suite of models. Working Paper No. 471.

[CR13] Coibion O, Gorodnichenko Y (2015). Information rigidity and the expectations formation process: A simple framework and new facts. The American Economic Review.

[CR14] Coibion, O., Dimitris, G., Gorodnichenko, Y., & Weber, M. (2020). Forward guidance and household expectations. NBER Working Paper No. 26778.

[CR15] Cornand C, Heinemann F, Duffy J (2014). Experiments on monetary policy and central banking. Experiments in macroeconomics. Research in experimental economics 17.

[CR16] Cornand C, M’baye C (2018). Does inflation targeting matter?. An Experimental Investigation. Macroeconomic Dynamics.

[CR17] Cornand C, Hubert P (2020). On the external validity of experimental inflation forecasts: A comparison with five categories of field expectations. Journal of Economic Dynamics and Control.

[CR18] Constncio, V. (2017). Developing models for policy analysis in central banks. In Annual Research Conference, Frankfurt am Main.

[CR19] Dorich, J., Johnston, M., Mendes, R., Murchison, S., & Y. Zhang (2013). ToTEM II: An updated version of the Bank of Canada’s quarterly projection model. Technical Report No. 100. Bank of Canada.

[CR20] Duffy, J. (2012). Macroeconomics: A Survey of Laboratory Research. University of Pittsburgh Working Paper No. 334.

[CR51] Evans GW, Honkapohja S (1999). Learning dynamics. Handbook of macroeconomics.

[CR21] Ferrero, G., & A. Secchi (2010). Central Banks’ Macroeconomic Projections and Learning. Bank of Italy Working Paper No. 782.

[CR50] Fischer S (1977). Long-term contracts, rational expectations, and the optimal money supply rule. Journal of Political Economy.

[CR22] Gabaix X (2014). A sparsity-based model of bounded rationality. The Quarterly Journal of Economics.

[CR23] Goodhart C, Lim WB (2011). Interest rate forecasts: A pathology.

[CR24] Goy, G., Hommes, C., & Mavromatis, K. (2020). Forward guidance and the role of central bank credibility under heterogeneous beliefs. *Journal of Economic Behavior & Organization*.

[CR25] Hommes C, Massaro D, Weber M (2019). Monetary policy under behavioral expectations: Theory and experiment. European Economic Review.

[CR26] Hommes C, Massaro D, Salle I (2019). Monetary and fiscal policy design at the zero lower bound: Evidence from the lab. Economic Inquiry.

[CR27] Hubert P (2014). FOMC forecasts as a focal point for private expectations. Journal of Money, Credit and Banking.

[CR28] Jain, M. & C. Sutherland. (2018). How do Central-Bank projections and forward guidance influence private sector forecasts? Staff Working Papers 18-2, Bank of Canada.

[CR29] Kamber G, McDonald C, Sander N, Theodoridis K (2015). A structural forecasting model for New Zealand: NZSIM.

[CR30] Kool CJ, Thornton D (2015). How effective is central bank forward guidance?. Federal Reserve Bank of St. Louis.

[CR31] Kravik EM, Yasin M (2019). Navigating with NEMO.

[CR32] Kryvtsov, O. & L. Petersen (2013). Expectations and monetary policy: Experimental evidence, Bank of Canada Working Paper 2013-44.

[CR33] Kryvtsov, O., & L. Petersen (2020). Central bank communication that works: Lessons from lab experiments. Journal of Monetary Economics.

[CR34] Lucas R (1972). Expectations and the neutrality of money. Journal of Economic Theory.

[CR52] Mackowiak B, Wiederholt M (2009). Optimal sticky prices under rational inattention. American Economic Review.

[CR35] Malmendier U, Nagel S (2016). Learning from inflation experiences. The Quarterly Journal of Economics.

[CR36] Mauersberger, F. (2017). Monetary policy rules in a non-rational world: A macroeconomic experiment. Columbia Economics Discussion Paper Series No. 161701.

[CR37] Mazzotta M, Opaluch J (1995). Decision making when choices are complex: A test of Heiner’s hypothesis. Land Economics.

[CR38] Marimon R, Sunder S (1993). Indeterminacy of equilibria in a hyperinflationary world: Experimental evidence. Econometrica.

[CR39] McCaw S, Ranchhod S (2002). The Reserve Bank’s forecasting performance.

[CR40] Mirdamadi, M. & L. Petersen (2018). Macroeconomic literacy and expectations. Working Paper.

[CR41] Mokhtarzadeh, F. & L. Petersen (2015). Coordinating expectations through central bank projections. Working paper. 10.1007/s10683-020-09684-6 PMC8550474 34720683

[CR42] Monti, F. (2018). The bank of England’s forecasting platform. In Seventh BIS research network meeting on “Pushing the frontier of central banks’ macro-modelling”.

[CR43] Pfajfar D, Santoro E (2010). Heterogeneity, learning and information stickiness in inflation expectations. Journal of Economic Behavior and Organization.

[CR44] Pfajfar D, Z̆akelj B (2014). Experimental evidence on inflation expectation formation. Journal of Economic Dynamics and Control.

[CR45] Pfajfar, D. & B. Z̆akelj (2016). Inflation expectations and monetary policy design: Evidence from the laboratory. Macroeconomic dynamics. 1–41.

[CR46] Rholes, R., & Petersen, L. (2020). Should central banks communicate uncertainty in their projections? *Journal of Economic Behavior & Organization*. Forthcoming.

[CR47] Simon HA (1959). Theories of decision-making in economics and behavioral science. The American Economic Review.

[CR100] Sims CA (2003). Implications of rational inattention. Journal of Monetary Economics.

[CR48] Turner J (2006). fAn assessment of recent Reserve Bank forecasts. Reserve Bank of New Zealand Bulletin.

[CR49] Walsh CE (2010). Monetary theory and policy.

