# Supplementary material for: Coordinating expectations through central bank projections
Source: Exp Econ. 2020 Nov 24;24(3):883–918. doi: 10.1007/s10683-020-09684-6 (PMC8550474; doi:10.1007/s10683-020-09684-6)
Supplement: Supplementary file 1 — Supplementary material 1 (pdf 3954 KB) [file 10683_2020_9684_MOESM1_ESM.pdf]

For Online Publication: Coordinating expectations through central bank projections - Appendix[1]

Fatemeh Mokhtarzadeh Luba Petersen

June 2020

This online appendix includes experimental instruction and supplementary results associated with Mokhtarzadeh and Petersen (2020) "Coordinating expectations through central bank projections".

[1] † Petersen: Department of Economics, Simon Fraser University, 8888 University Drive, Burnaby, BC, V5A 1S6, Canada, lubap@sfu.ca. Mokhtarzadeh: Department of Economics, University of Victoria, Victoria, BC, V8P 5C2, Canada

Appendix A– Experimental instructions

Below are instructions for the NoComm, IRProj, DualProj and ADProj treatments. Across treatments, the instructions differed only in the presentation of information about the projection and in the description of the user interface.

EXPERIMENTAL STUDY OF ECONOMIC DECISION MAKING

Welcome! You are participating in an economic experiment at CRABE Lab. In this experiment you will participate in the experimental simulation of the economy. If you read these instructions carefully and make appropriate decisions, you may earn a considerable amount of money that will be immediately paid out to you in cash at the end of the experiment.

Each participant is paid CAN $7 for attending. Throughout this experiment you will also earn points based on the decisions you make. Every point you earn is worth $0.50. We reserve the right to improve the show up fee in your favour if average payoffs are lower than expected.

During the experiment you are not allowed to communicate with other participants. If you have any questions, the experimenter will be glad to answer them privately. If you do not comply with these instructions, you will be excluded from the experiment and deprived of all payments aside from the minimum payment of CAN $7 for attending.

The experiment is based on a simple simulation that approximates fluctuations in the real economy. Your task is to serve as private forecasters and provide real–time forecasts about future output and inflation in this simulated economy. The instruction will explain what output, inflation, and the interest rate are and how they move around in this economy, as well as how they depend on forecasts. You will also have a chance to try it out for 4 periods in a practice demonstration.

In this simulation, households and firms (whose decisions are automated by the computer) will form forecasts identically to yours. So to some degree, outcomes that you will see in the game will depend on the way in which all of you form your forecasts. Your earnings in this experiment will depend on the accuracy of your individual forecasts.

Below we will discuss what inflation and output are, and how to predict them. All values will be given in basis points, a measurement often used in descriptions of the economy. All values can be positive, negative, or zero at any point in time.

How the economy evolves

You will submit forecasts for the next period's inflation and output, measured in basis points:
1% = 100 basis points
3.25% = 325 basis points
-0.5% = -50 basis points
-4.8% = -480 basis points

Read aloud: These are just a handful of examples. You can submit any forecast you wish, positive or negative or zero, but please only submit integers.

The economy consists of four main variables:

- Inflation, Output, Interest Rate, Shocks

Read aloud: Your goal in this experiment is to forecast future output and inflation as correctly as possible. We will now explain the factors that influence output and inflation and the relationships between the different variables in the economy. We begin with 'Shocks'. Shocks are simply changes to the amount of output consumers in the economy wish to purchase. They are changing every period and are influenced by a random component and past shocks. More precisely, the shocks that you observe will follow the process specified in your instructions. Intuitively, you can think of the shocks as weather shocks. Over the long run, the weather has no effect on how much consumers want to buy. However, from day to day, there may be random changes to the weather. You can think of a positive shock as unexpectedly nice weather. When the weather is especially nice, consumers are spending more time out of their homes and increasing their expenditures (for example, buying ice cream, going out for a nice dinner, going to the beach). A negative shock can be thought of unexpectedly terrible weather, where no one wants to leave their homes, causing expenditures to be relatively low. Gradually, the shocks, like weather, will revert back to their long-run levels. As the shocks dissipate, new random events occur that will make consumers want to increase or decrease their expenditures. The shocks to the economy will have a precise value and will be displayed on your screen.

At any time, t, the values of these variables will be calculated as follows:

$Shock_t = 0.57(Shock_{t-1}) + Random\ Component_t$

- The random component is 0 on average.
- Roughly two out of three times the shock will be between -138 and 138 basis points.
- 95% of the time the shock will be between -276 and 276 basis points.

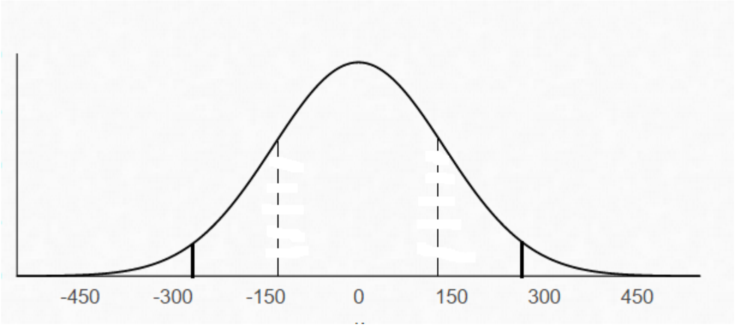

E.g.

$$\begin{aligned} Shock_1 &= 30 \\ Shock_2 &= 30 \times 0.57 + New\ Draw \\ &= 17.1 + (30) \\ &= 47.1 \\ Shock_2 &= 17.1 + (-150) \\ &= -132.9 \end{aligned}$$

Read aloud: While these shocks have been drawn from a random distribution, we have pre-selected sequences that we are interested in studying. Now we will discuss precisely how the economy evolves. Each period, you and the other forecasters will be submitting your beliefs about the following period's output and inflation. The median of each of the forecasts will be

employed as the aggregate forecast in the given period and play an important role in determining the current level of output and inflation. The median, rather than the average forecast, is used so that a small number of subjects cannot have a significant effect on the economy. However, if the majority of forecasters expect relatively high inflation or output in the future, the aggregate forecasts will be higher. Inflation is determined largely by aggregate forecasts about future inflation. The idea behind this is simple: If the professional forecasters communicate to the public that inflation is likely to rise in the future, consumers will spend more immediately to avoid paying relatively higher prices in the future. This increase in demand will cause prices to start rising, ie. current inflation will increase. Current output will also have a small positive effect on current inflation.

The amount of output that will be produced in any given period will also depend on the median forecaster's expectation, formed that period, about future output and inflation. If the forecasters predict that the future economy will be producing more output and there will be more inflation, consumers will want to spend more in the current period. Firms will then produce more to meet consumer demand. Likewise, positive shocks to consumer demand will have a positive effect on how much will be produced. Increases in the nominal interest rate will make it more expensive for consumers to borrow and will create more incentive for them to save. With higher interest rates, consumers will decrease their demand for goods, leading to lower production, which will indirectly reduce inflation.

Finally, the central bank in this economy will be adjusting the nominal interest rate to keep output and inflation as close to zero as possible. As inflation increases, the central bank will increase interest rates more than one-for-one with inflation. The central bank will also respond, though less aggressively, to output. The increase in the nominal interest rate has a direct negative effect on consumer demand and production, and an indirect negative effect on inflation. Importantly, you will not observe the current interest rate when you are forming your forecast about the following period's inflation and output. After you submit your forecasts, the computer will simultaneously solve for the current period's inflation, output and inflation taking into consideration the median forecasts and the realized shock. It is also important for you to realize that, even though the central bank is aiming for zero inflation and output, it will rarely accomplish that because of the random shocks that are occurring each period and the public's expectations. However, the economy will be kept relatively more stable as a consequence of the central bank's reaction to inflation and output.

How the economy evolves:

$$Inflation_t = 0.989(Median\ forecast\ of\ Inflation_{t+1}) + 0.13(Output_t)$$

$$Output_t = Median\ forecast\ of\ Output_{t+1} + Median\ forecast\ of\ Inflation_{t+1} - Interest\ Rate_t + Shock_t$$

$$Interest\ Rate_t = 1.5(Inflation_t) + 0.5(Output_t)$$

- The Central Bank sets the target for output and inflation at zero. In order to achieve the target it will adjust the interest rate and in some cases this means the interest rate can become negative.
- Expectations are self-fulfilling in this economy. If the median subject forecasts higher

inflation and output in the future, both inflation and output will grow higher in the current period. Similarly, median forecasts of negative inflation and output will cause the economy to recede in the current period.

DEPENDING ON THE PROJECTION TREATMENT, SUBJECTS RECEIVED ONE OF THREE PIECES OF INFORMATION ABOUT THE PROJECTION(S):

- **IRProj:** The Central Bank will make a five–period projection each period about the future level of the interest rate. It is important to remember that the projections are simply a forecast and not a promise. The Central Bank uses the model and current and expected future shocks to form its projections. In particular, it predicts that interest rates will return to zero in the near future.
- **DualProj:** The Central Bank will make a five–period projection each period about the future levels of the inflation and output. It is important to remember that the projections are simply a forecast and not a promise. The Central Bank use the model and the current and expected future shocks to form its projections. In particular, it predicts that the economy will return to zero levels of inflation and output in the near future.
- **ADProj:** The Central Bank will make a five–period projection each period about the future levels of the inflation and output. It is important to remember that the projections are simply a forecast and not a promise. The Central Bank use a combination of the model, current and expected future shocks as well as the previous period's output and inflation to form its projections. In particular, it predicts that the economy will return to zero levels of inflation and output in the near future.

Score

Your score will depend on the accuracy of your forecasts. The absolute difference between your forecasts and the actual values for output and inflation are your absolute forecast errors.

- Absolute Forecast Error= absolute(Your Forecast - Actual Value)
- Total Score = $0.30(2^{-0.01(AbsoluteForecastErrorforOutput)})$+$0.30(2^{-0.01(AbsoluteForecastErrorforInflation)})$

The maximum score you can earn each period is 0.60. Your score will decrease as your forecast error increases. Suppose your forecast errors for each of output and inflation is:

1. 0 : Your score will be 0.6
2. 50: Your score will be 0.42
3. 100: Your score will be 0.30
4. 200: Your score will be 0.15
5. 300: Your score will be 0.075
6. 500: Your score will be 0.02
7. 1000: Your score will be 0
8. 2000: Your score will be 0

During the experiment, your main screen will display information that will help you make forecasts and earn more points.

At the top left of the screen, you will see your subject number, the current period, time remaining, and the total number of points earned. Below that you will see you will also see

three history plots. The top history plot displays past interest rates and shocks, *as well as the Central Bank projection of interest rates (IRPRoj)*. The second plot displays your past forecast of inflation and realized inflation levels, *and the Central Bank projection (DualProj and ADProj)*. The final plot displays your past forecasts of output and realized output levels, *and the Central Bank projection (DualProj and ADProj)*.

The difference between your forecasts and the actual realized levels constitutes your forecast errors. Your forecasts will always be shown in blue while the realized value will be shown in red. *The central bank forecast will be shown in green (IRProj, DualProj, ADProj)*. You can see the exact value for each point on a graph by placing your mouse at that point.

When the first period begins, you will have 65 seconds to submit new forecasts for the next period's inflation and output levels. You may submit both negative and positive forecasts. Please review your forecasts before pressing the SUBMIT button. Once the SUBMIT button has been clicked, you will not be able to revise your forecasts until the next period. You will earn zero points if you do not submit the two forecasts. After the first 9 periods, the amount of time available to make a decision will drop to 50 seconds per period. You will participate in two sequences of 30 periods, for a total of 60 periods of play. Your score, converted into Canadian dollars, plus the show up fee will be paid to you in cash at the end of the experiment.

Appendix B–Aggregate expectations and participant incentives

The usage of the median forecast as the aggregate forecast is not entirely innocuous. Suppose a subject believes she is the median forecaster. She maximizes her payoffs by making a statistically accurate forecast of a fixed point of the stable system of equations governing the economy. Thus, she would have the incentive to report true beliefs knowing that her forecast influences the aggregate state of the economy. A participant, interacting in groups of 7, has a small but positive probability of being the median forecaster for each of her two forecasts. If, in Period t, she anticipates being the median forecaster for either of her forecasts, she may have the incentive to adjust their forecast about Period $t+1$ to influence Period t output gap and inflation, and consequently, the accuracy of their forecasts formed in Period $t-1$. However, being only one of seven subjects, she is unlikely to be pivotal and, thus, have less incentive to adjust her forecasts to improve the accuracy of her previous forecast. Moreover, there is limited scope to influence the aggregate forecast without losing one's position as the median forecaster. We also never reveal to participants whether they were the median forecasters, thus reducing the likelihood that they identify themselves as being pivotal in the past.

Our design only deviates from incentive compatibility from the perspective of participant A to the extent that A believes that other participants' forecasts will be dispersed, thus leaving her the potential to influence the median. If A believes that others are forecasting relatively tightly, she should expect that she cannot influence the median. Thus, she has no incentive to deviate from her prediction of the future. She maximizes her payoffs by reporting her true beliefs of the subsequent period. If she does not anticipate that she will be the median forecaster, she should form expectations based on the anticipated forecasts of the median forecaster(s). 'Best-responding' to the median forecaster would maximize her payoffs.

The only incentive issue posed with this design is, as mentioned earlier, is if a subject were to submit a period t forecast of t+1 in order to increase the accuracy of her forecast submitted in period t-1. The median rule makes this incentive small relative to the strong incentive to forecast accurately about the next period. This problem would instead be fully solved if, instead, two groups of subjects where one made forecasts in even periods, the other in odd periods. This alternative design would require a smaller number of participants determining the state of the economy each period (which has the potential to introduce additional volatility) or double the number of participants. Given that our incentive scheme's deviation from perfect incentive compatibility is, at most, small and negligible under rational theory-consistent conditions, we believe that the current approach was preferable for the research questions we are interested in, especially since this enhances our comparability to previous related literature.

Employing the median forecasts as the aggregate forecasts is preferable to the alternative. In related LtFEs, using the mean forecasts as the aggregate expectations can lead to unstable dynamics driven by outlier subjects and greater incentive to bias forecasts. Moreover, the incentive scheme and experiment are designed so that participants do not submit random forecasts or a constant value (e.g., zero), but their best guess of how output and inflation will evolve based on aggregate expectations, shocks, and the data-generating process.

We finally note that experimental economists routinely use the term *incentive-compatible*

mechanism to refer to the BDM and random incentive schemes, even though it is well-known that these will only elicit true preferences if subjects are expected utility maximizers – see the discussion in Holt (1986) and Karni and Safra (1987). In a similar vein, our incentive scheme is *incentive compatible* if subjects satisfy rational economic theory. Given the different ways that *incentive compatible* is used in experimental economics, we do not use this term when discussing our design.

Appendix C–Supplementary results and discussion

C.1. Aggregate outcomes

In this section, we consider the effects of central bank projections on aggregate macroeconomic variables. Our analysis begins by considering how the dynamics of output, inflation, and nominal interest rates respond to different forms of communication. We estimate the orthogonalized impulse responses of output, inflation, and nominal interest rates to a one-standard deviation shock to aggregate demand. The results for Repetition 2 are presented in Figure 1 by shock sequence, ordered from least to most volatile sequences. The heavy solid lines indicate the estimated REE predictions, while the thin solid lines denote the estimated impulse response functions in the NoComm treatment. Figure 2 and Figure 3 plots the time series of output and inflation by session and repetition.

The initial response of output to the demand shock in the NoComm treatment is rather consistent with the REE prediction. However, output sluggishly declines thereafter and by the fourth period output becomes negative before returning to the steady state. Inflation follows a noticeably different transition path from the REE prediction. On impact of the aggregate demand shock, inflation in the NoComm treatment exhibits a relatively muted response in most sessions. In those sessions, inflation then rises for two additional periods before beginning to trend back toward the steady state. The hump-shaped pattern of inflation is indicative of an Adaptive(2) forecasting model where the aggregate expectation of $t + 1$ places significant positive weight on inflation from period $t - 2$.

Central bank projections have varying effects on the transition paths of output and inflation. The estimated impulse response functions are presented for the IRProj as blue dashed lines, the DualProj as short red dashed lines, and the ADProj as green long dash–dot–long dash lines. Generally, all three types of projections have a similar effect on output dynamics for low variability shocks. As the shocks become more variable, nominal interest rate and adaptive dual projections are associated with a greater contractionary overshooting effect of output, suggestive of a larger backward-looking nature of forecasts consistent with behavior in the NoComm treatment.

The effects of central bank communication are more stark when we consider the estimated responses of inflation. Rational projections lead to a response of inflation that is considerably more inline with the REE prediction. Inflation is more consistently monotonically converging back to the steady state, and the hump-shaped pattern observed in the NoComm treatment is largely eliminated. We observe noticeable heterogeneity across sequences in the estimated

impulse response functions of IRProj sessions. The impulse response functions tend to track the timing of the REE prediction better when the shock volatility is relatively low. However, for relatively more volatile shock sequences such as Sequences 4 and 6, the reactions of inflation under IRProj are more sluggish and exhibit timing similar to that of the NoComm treatment. In other words, for greater shock volatility, central bank projections of nominal interest rates do not considerably alter forecasting heuristics and inflation dynamics.

The dynamics of inflation in the ADProj treatment are rather unusual. In all sequences, inflation significantly overshoots the REE prediction, and remains high thereafter as the shocks dissipate. The persistently high inflation is associated with ADProj subjects' over-reaction to lagged innovations. In half of the sessions, inflation becomes negative as a consequence of subjects' backward-looking forecasting heuristics combined with an aggressive response of monetary policy to high inflation.

Figure 1: Estimated impulse responses of endogenous variables to 113 basis points shock

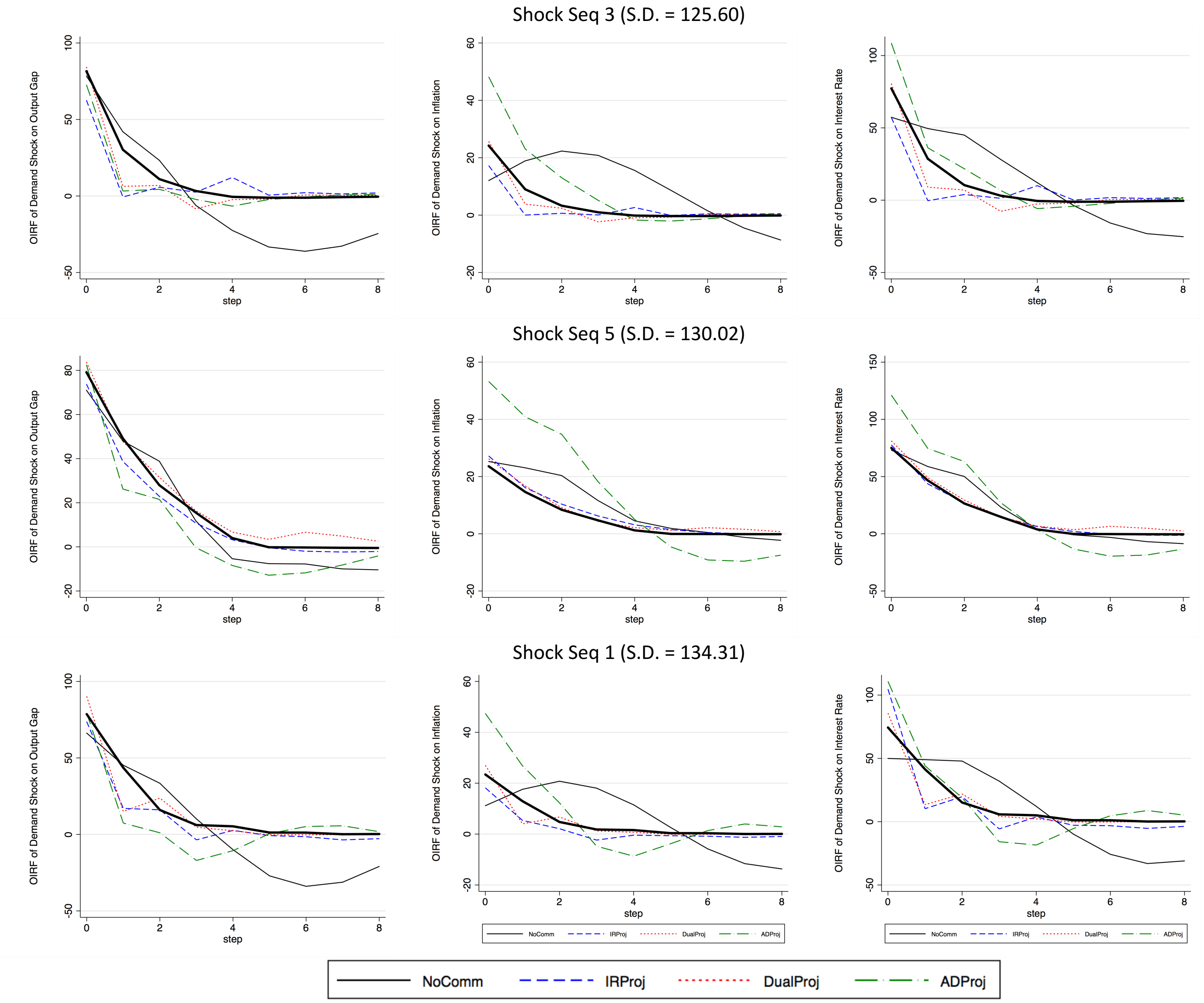

Figure 1: Estimated impulse responses of endogenous variables to 113 basis point shock

*The figure shows the impulse responses of the variables to one standard deviation of the shock in basis points.

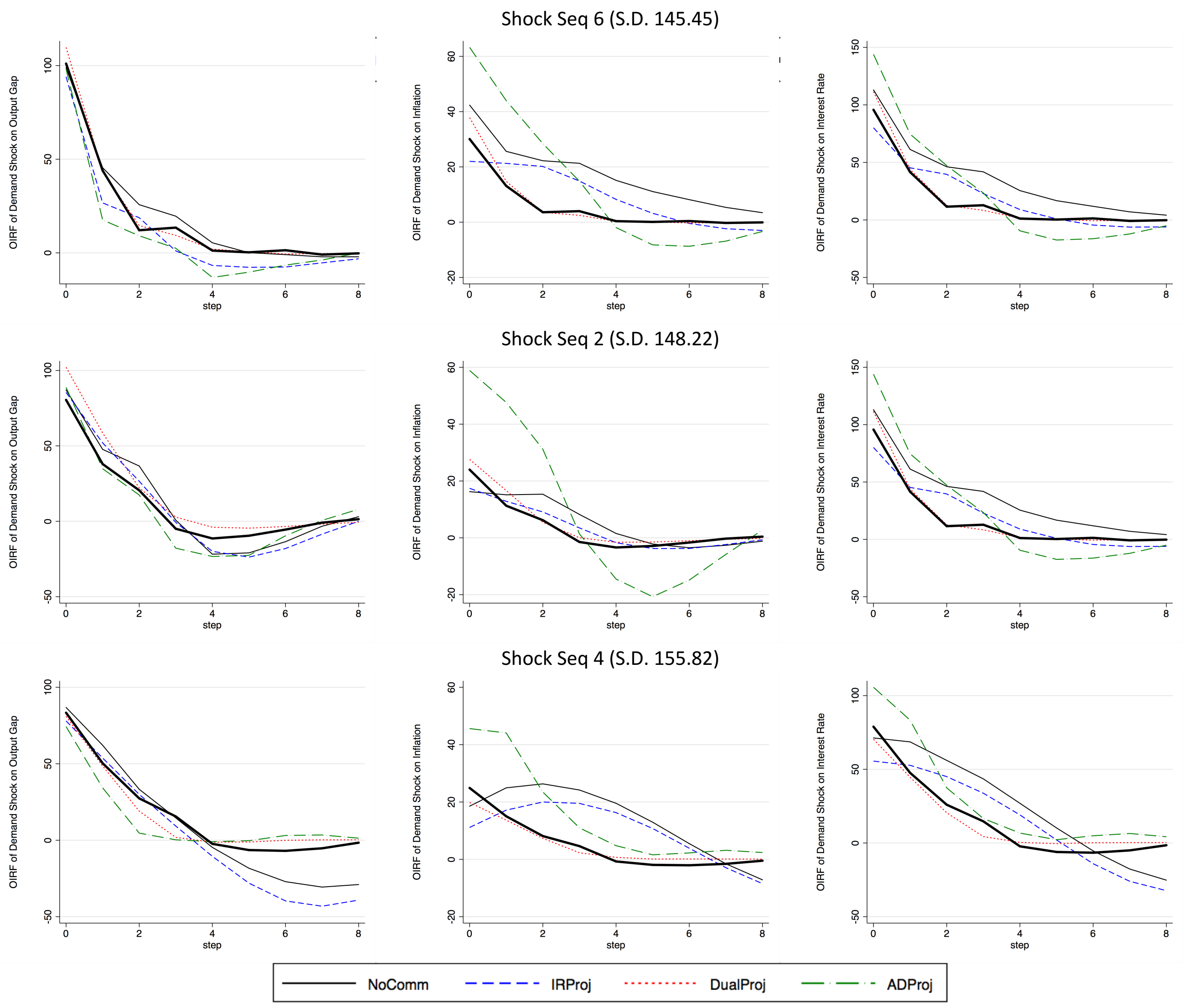

Figure 2: Time series of the output gap by session and repetition

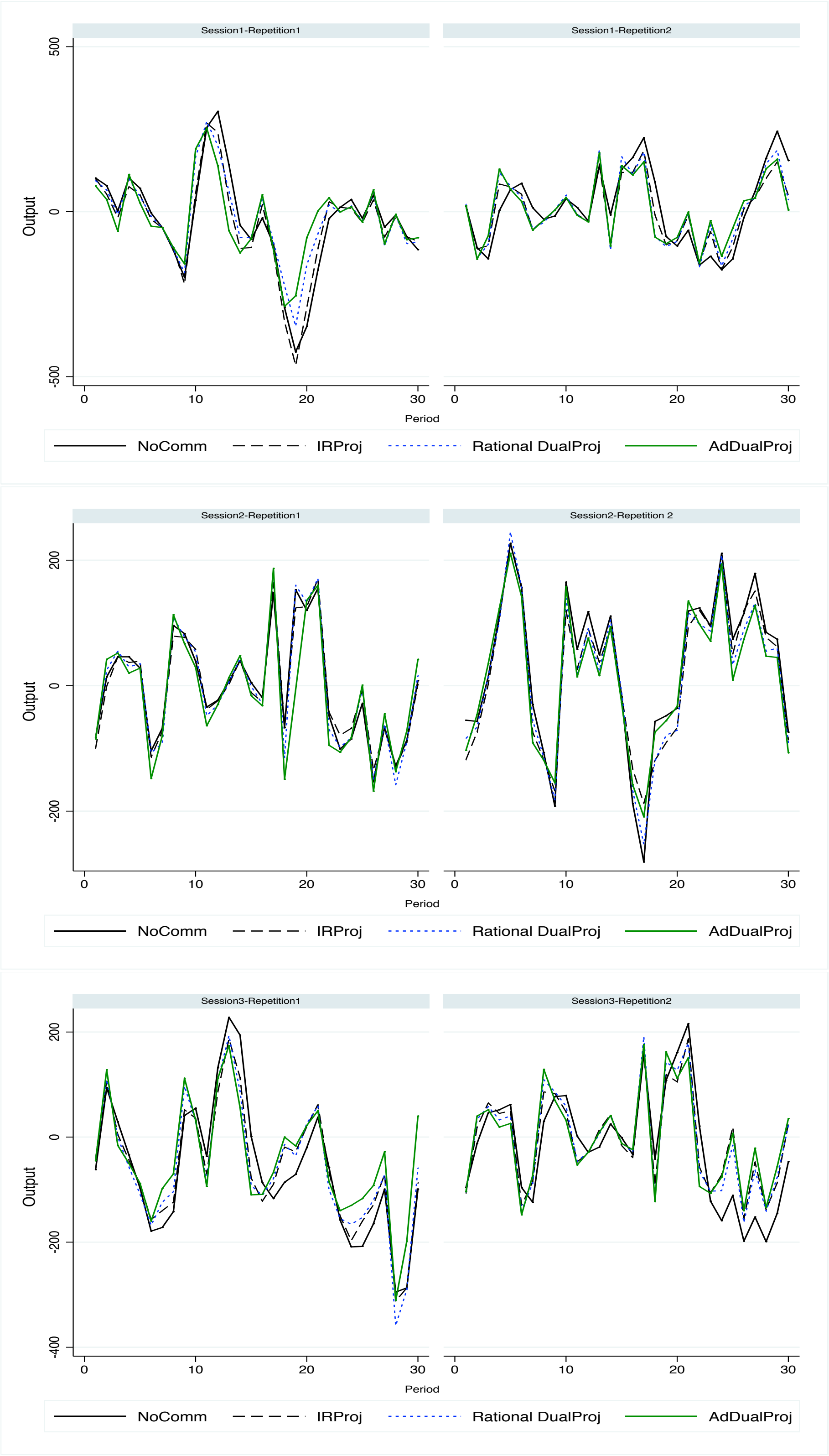

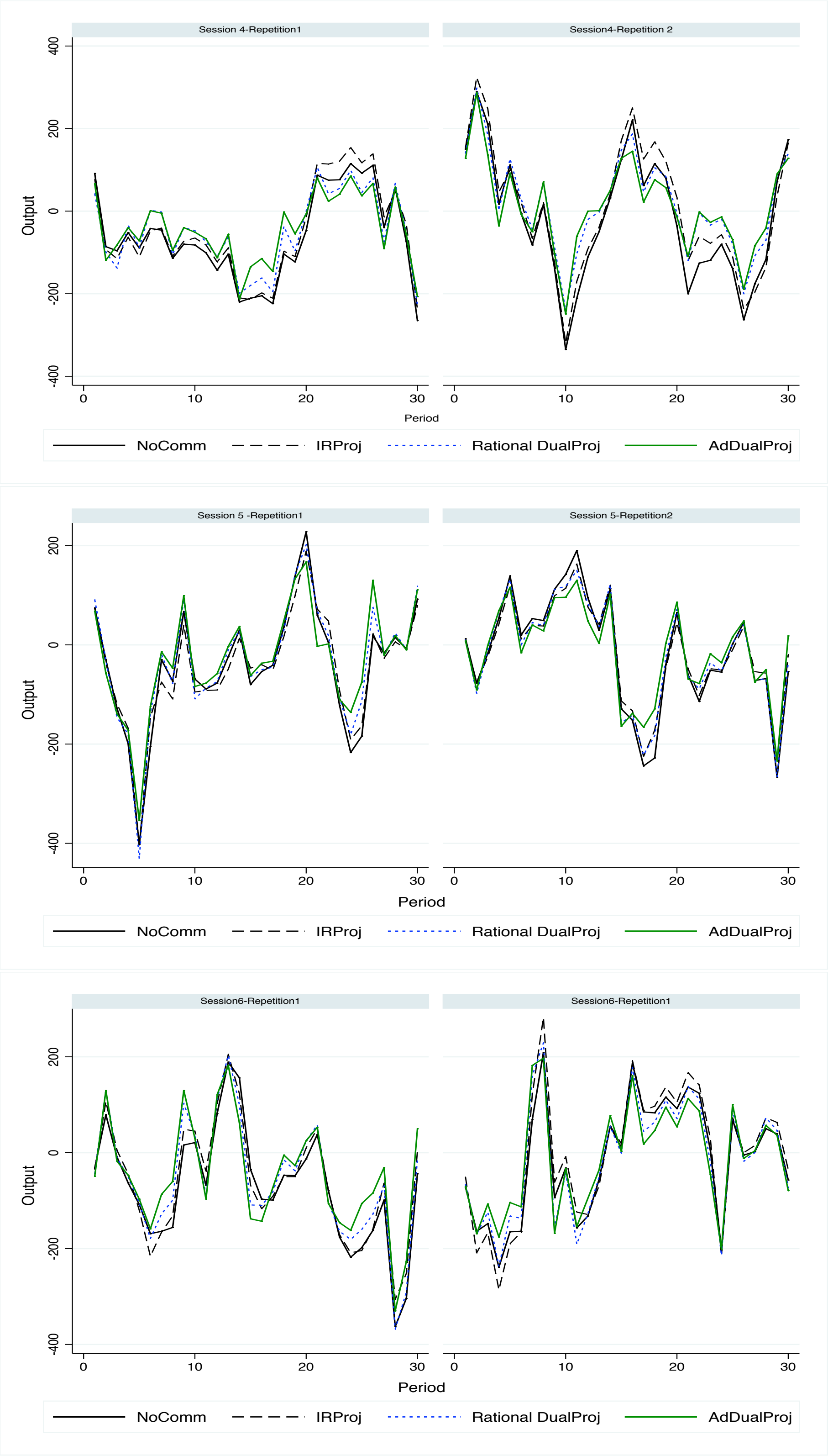
Session 4-Repetition1
Session4-Repetition 2
Output
Period
NoComm
IRProj
Rational DualProj
AdDualProj
Session 5 -Repetition1
Session 5-Repetition2
Output
Period
NoComm
IRProj
Rational DualProj
AdDualProj
Session6-Repetition1
Session6-Repetition1
Output
Period
NoComm
IRProj
Rational DualProj
AdDualProj

Figure 3: Time series of the inflation by session and repetition

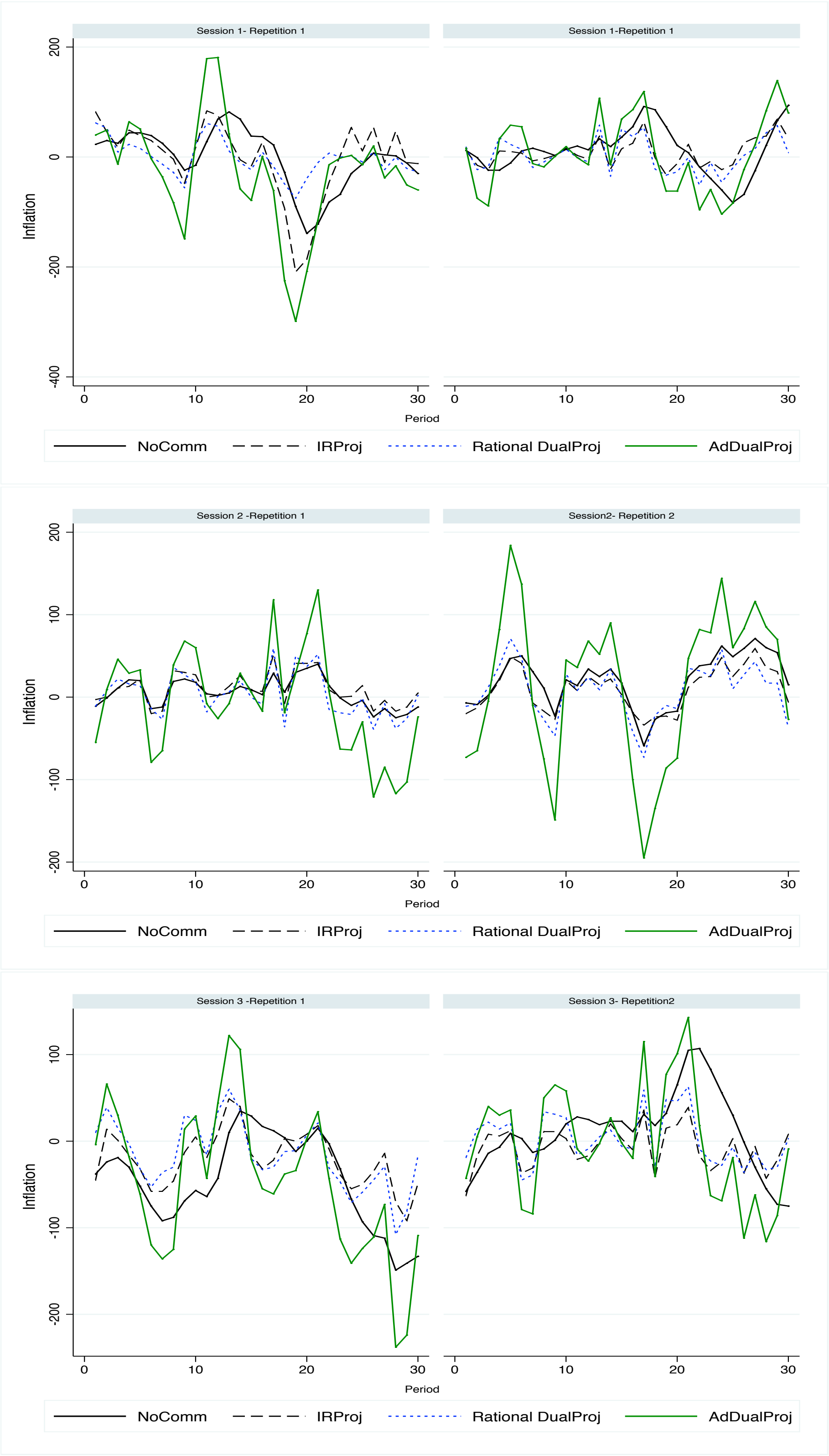

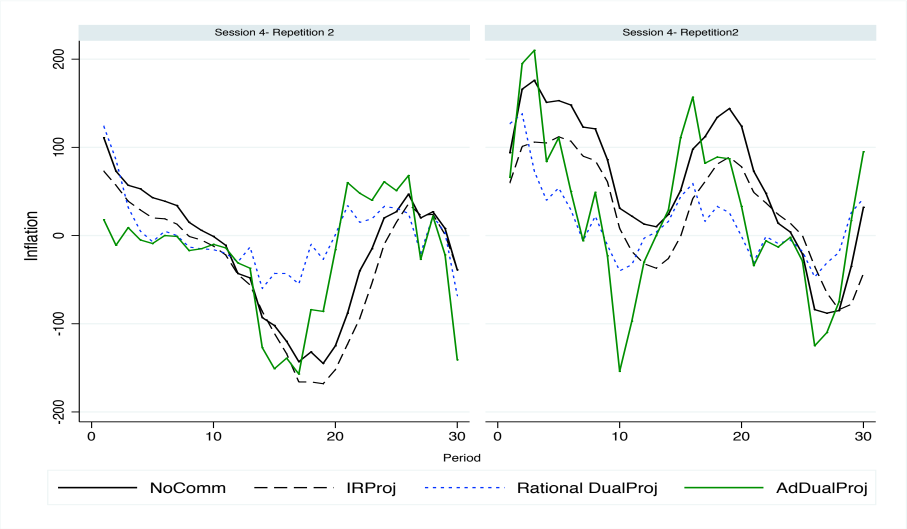
Session 4- Repetition 2
Session 4- Repetition2
Inflation
200
100
0
-100
-200
0
10
20
30
Period
NoComm
IRProj
Rational DualProj
AdDualProj

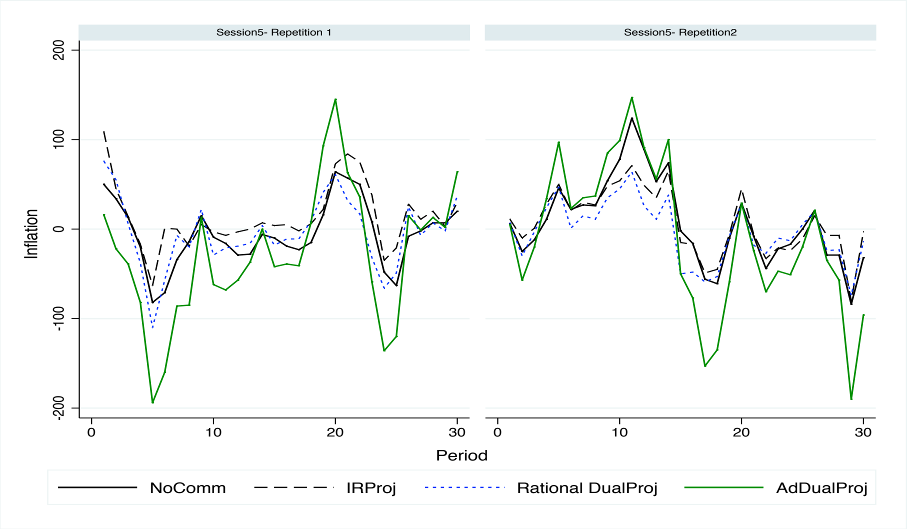
Session5- Repetition 1
Session5- Repetition2
Inflation
200
100
0
-100
-200
0
10
20
30
Period
NoComm
IRProj
Rational DualProj
AdDualProj

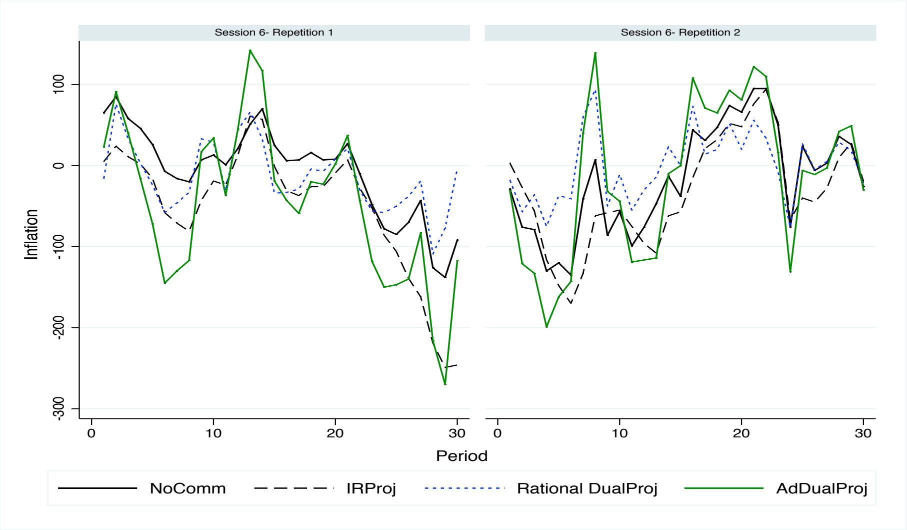
Session 6- Repetition 1
Session 6- Repetition 2
Inflation
100
0
-100
-200
-300
0
10
20
30
Period
NoComm
IRProj
Rational DualProj
AdDualProj

C.2. Recursive learning and projections

Ferrero and Secchi (2010) consider the impact of the publication of CB projections on the dynamic properties of an economy where private agents have incomplete information and form expectations using recursive learning algorithms (Marcet and Sargent, 1989; and Evans and Honkapohja, 2001). As in our experiment, they assume that the short-term nominal interest rate responds linearly to deviations of inflation and output from their target level, and that the CB assumes agents form expectations according to the REE solution. Ferrero and Secchi find that nominal interest rate projections shrink the set of interest rate rules associated with stable equilibria under learning and slows down learning. This is a consequence of the CB failing to take into account systematic errors private agents form as they are learning, leading to a weak positive feedback of monetary policy, and a system that is more vulnerable to self-fulfilling expectations. By contrast, publication of inflation and output projections reduces the inflationary bias in agents' expectations, expanding the set of policy rules that would allow for stability under learning.

Proposition 1 (Ferrero and Secchi, 2010). Let $\sigma\phi_x + \kappa\sigma\phi_\pi + 1 \neq 0$. Given (i) the data-generating process, where (ii) at time t the central bank publishes the time $t+1$ interest rate projection consistent with the REE and (iii) recursive learning private agents assign weight $0 \leq (1-\lambda_1) \leq 1$ to these projections[2], revealing the interest rate path makes the condition for stability under learning more stringent than under no announcement. In particular, the necessary and sufficient conditions for E-stability of the REE is:

$$\phi_\pi > \frac{2}{1+\lambda_1} - \frac{1-\beta}{\kappa}\phi_x.$$

The greater the weight assigned to the interest rate projection, the more the central bank must respond to concurrent inflation and output in order to maintain E-stability of the REE. Figure 4 compares the regions of E-stability in the (γ_x, γ_π) space under communication and no communication of the central bank's REE interest rate projection assuming full internalization of the projection, $1-\lambda_1 = 1$. Given the parameterization of our laboratory experiments, the REE is e-unstable when at least a fraction $1-\lambda_1 = 0.703$ of subjects fully employ the interest rate projection as their implicit forecast for interest rates.

[2] Alternatively, it can be assumed that a fraction of agents $1-\lambda_1$ fully internalize the central bank's projection while the remaining agents continue to forecast using their recursive learning model.

Figure 4: E-stability under recursive learning and no-announcement $(1-\lambda_1)=0$ and under a fully-internalized announcement of the interest rate path, $(1-\lambda_1)=1$.

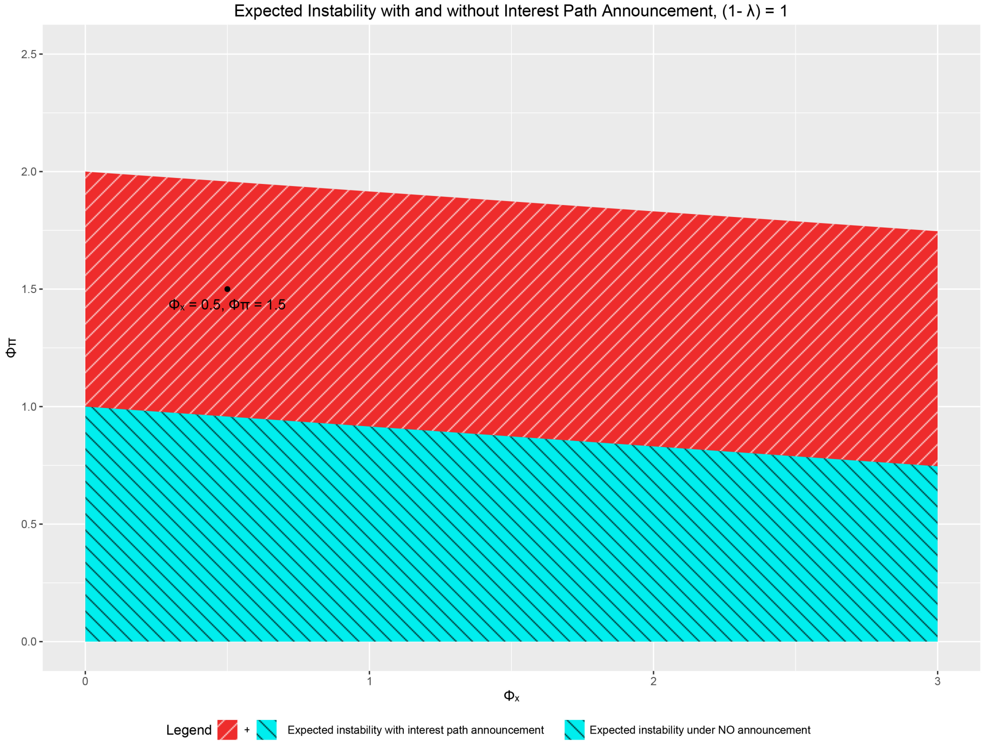

Proposition 2 (Ferrero and Secchi, 2010). Let $\sigma\phi_x + \kappa\sigma\phi_\pi + 1 \neq 0$. Given (i) the data-generating process, where (ii) at time t the central bank publishes the time $t+1$ output and inflation projections consistent with the REE and (iii) recursive learning private agents assign weight $0 \leq (1-\lambda_2) \leq 1$ to these projections, revealing the projected paths makes the condition for stability under learning less stringent than under no announcement. In particular, the necessary and sufficient conditions for E-stability of the REE is:

$$\phi_\pi > \frac{2\lambda_2(\beta\lambda_2+1)\kappa\sigma - (\beta^2\lambda_2-1)(\lambda_2-1)}{(\beta\lambda_2+1)(1+\lambda_2)\kappa\sigma} - \frac{(1-\beta^2\lambda_2)}{(\beta\lambda_2+1)\kappa}\phi_x.$$

The greater the weight assigned to the dual projections, the less the central bank must respond to concurrent inflation and output in order to maintain E-stability of the REE. Figure 5 compares the regions of E-stability in the (γ_x, γ_π) space under communication and no communication of the central bank's REE output and inflation projections assuming minimal internalization of the projection, $1-\lambda_1 = 0.1$. Given the parameterization of our laboratory experiments, the REE is E-stable under recursive least squares learning irrespective of the number of subjects that employ the central bank's macroeconomic projections.

[Figure 5:](#) E-stability under recursive learning and no-announcement $(1-\lambda_2) = 0$ and under a minimally-internalized announcement of the interest rate path, $(1 - \lambda_2) = 0.1$.

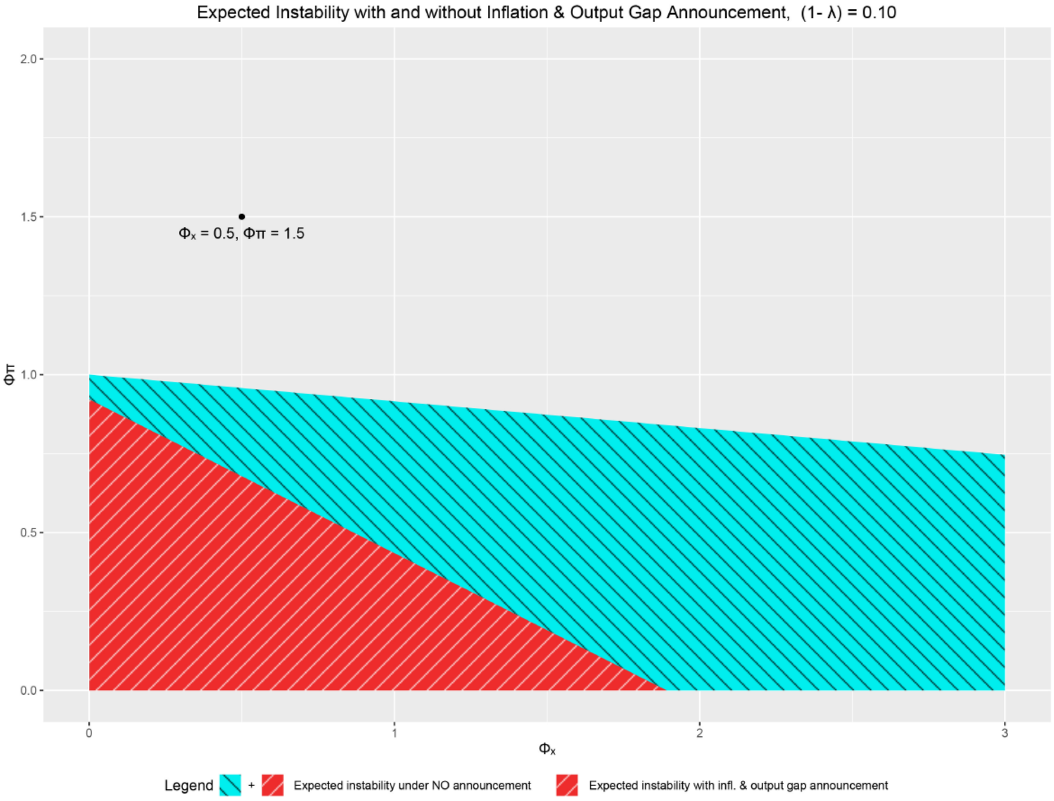

Given the data–generating process given by Equations (1)-(4) in our paper, where at time t the central bank publishes the time $t+1$ interest rate projection consistent with the REE and recursive learning private agents assign weight $0 \leq (1-\lambda_1) \leq 1$ to these projections revealing the interest rate path makes the condition for stability under learning more stringent than under no announcement.[3] Given the parameterization of our laboratory experiments, the REE is e-unstable when at least a fraction $1 - \lambda_1 = 0.703$ of subjects fully employ the interest rate projection as their implicit forecast for interest rates.

On the other hand, if at time t the central bank publishes the time $t+1$ output and inflation projections consistent with the REE and recursive learning private agents assign weight $0 \leq (1-\lambda_2) \leq 1$ to these projections, revealing the projected paths makes the condition for stability under learning less stringent than under no announcement. Given our parameterization, the REE is E-stable under recursive least squares learning irrespective of the number of subjects that employ the central bank's macroeconomic projections.

Compared to those in the NoComm, the median DualProj forecasters formed expectations that were significantly more in line with the REE solution. We observe a similar pattern for the median IRProj forecasters in sequences with less variable shocks. However, in more volatile shock sequences, we do not observe significant improvement in forecasting towards the REE solution.

[3]Alternatively it can be assumed that a fraction of agents $1 - \lambda_1$ full internalize the central bank's projection while the remaining agents continue to forecast using their recursive learning model.

There are at least two possible explanations for why the IRProj sessions did not experience more severe instability. First, few IRProj subjects paid attention to the interest rate projection. An average of 7–13% of subjects in the IRProj treatment formed expectations that were within five basis points of the intended REE solution. This is far fewer subjects than necessary to generate instability. Under shock sequence 4, where deviation from REE was the greatest, the correlation between the median subject's expectations and the projection was the weakest (Spearman correlation coefficient for output = 0.07 with p=0.71, Spearman correlation coefficient for inflation was 0.47 with p=0.01). Second, our subjects were more informed about the data–generating process than the recursive learning agents in Ferrero and Secchi's model. The additional quantitative knowledge about the economy'sstructure may have mitigated the likelihood of instability. As Eusepi and Preston (2010) demonstrate, communicating the precise details of the central bank's policy is sufficient for anchoring private agents' expectations. We conducted a couple of sessions (not reported here) involving interest rate projections where subjects were only provided qualitative information about the economy's data–generating process. We find no noteworthy difference in the stability of our macroeconomic variables when subjects are less informed.

C.3. Sticky information models

Mankiw and Reis (2002) and Reis' (2006) models of inattention consider agents that obtain information infrequently due to costly information acquisition. When agents do receive information, they receive perfect information and are able to make optimal decisions. In the context of our experiment, this model would predict that agents would infrequently adjust their forecasts, but that their forecast errors would on average equal zero when they do adjust.

First, we note that the frequency of revision does vary considerably across subjects. Figure 6 presents the distribution of frequency of forecast updating across experienced subjects in each treatment. In the second repetition, the mean subject fails to update their output (inflation) forecast 3.6% (6.3%) of the time in NoComm treatment, 9% (12.3%) in the IRPRoj, 4% (6.7%) in the DualProj, and 4% (5.3%) in the ADProj treatments. The frequency of 'sticky information' is more than double in the IRProj than in the other treatments and occurs for both low and high variable shocks. There are considerably more subjects in the IRProj who fail to update their forecast frequently. For example, 15-20% of IRProj subjects update their output forecast less than 80% of the time. When subjects do update their forecast for the next period, they correctly forecast under 2% of the time. Forecast accuracy generally improves with almost all forms of projections and both low and high variability of shocks.[4] While we find evidence of substantial infrequent updating of forecasts by some subjects, especially in the IRProj treatment, their relative success at updating is very low.

To better understand what influences a subject not to update their forecast between two periods, we conduct a series of probit regressions where we evaluate the effects of past revisions, past forecast errors, absolute magnitude of shocks, treatment–specific dummies, and

[4] The exception is when subjects are forecasting inflation in the IRProj and ADProj treatments for low variability shocks. The proportion of accurate inflation forecasts drop from 1.02% in the NoComm treatment to 0.56% and 0.8% in the IRProj and ADProj treatments, respectively.

interactions of those treatment-specific dummies with the absolute magnitude of shocks. Table 1 reports our results by repetition for low and high variable shock sequences. Consistently, past revisions and larger forecast errors tend to be a strong positive predictor of revising in the following periods. The magnitude of the current shock does not have a consistent effect on subjects' likelihood of revising their forecast. We do, however, observe that nominal interest rate projections increase the likelihood a subject will fail to update their forecast. This effect is large and statistically significant in most of our specifications.

Subjects that do update their forecast do not usually update correctly. Less than 10% of output forecasts are within 10-basis points of the correct forecast, while 16-23% of inflation forecasts are within 10-basis points of the correct forecast. While the sticky information model captures the fact that some of our subjects fail to update their forecasts as new information arises, it fails to describe our subjects' inability to respond optimally to that information when they do update their forecasts.

Table 1: Effects of central bank projections on the likelihood of forecast revision - Treatment effects[I]

	Repetition 1				Repetition 2			
	$Pr(\Delta E_t x_{t+1}) = 0$		$Pr(\Delta E_t \pi_{t+1}) = 0$		$Pr(\Delta E_t x_{t+1}) = 0$		$Pr(\Delta E_t \pi_{t+1}) = 0$	
SD of shocks	Low	High	Low	High	Low	High	Low	High
$\Delta E_{t-1} x_t = 0$	0.405***	0.520***			1.058***	0.569***		
	(0.15)	(0.12)			(0.13)	(0.14)		
$\lvert E_{t-2}x_{t-1} - x_{t-1}\rvert$	-0.002	-0.002***			-0.001	-0.002**		
	(0.00)	(0.00)			(0.00)	(0.00)		
$IRProj$	0.396**	0.405**	0.307	0.062	0.426**	0.209	0.242	0.289
	(0.19)	(0.18)	(0.19)	(0.16)	(0.19)	(0.19)	(0.16)	(0.18)
$DualProj$	0.001	0.312*	0.085	-0.076	0.115	0.003	-0.187	-0.027
	(0.19)	(0.17)	(0.20)	(0.16)	(0.21)	(0.21)	(0.17)	(0.20)
$ADProj$	0.088	-0.146	-0.072	-0.226	0.029	0.308	-0.514***	0.075
	(0.20)	(0.19)	(0.20)	(0.16)	(0.21)	(0.20)	(0.20)	(0.19)
$\lvert\epsilon_t\rvert$	0.001	-0.001	-0.001	-0.002*	0.000	-0.001	-0.002*	-0.001
	(0.00)	(0.00)	(0.00)	(0.00)	(0.00)	(0.00)	(0.00)	(0.00)
$\lvert\epsilon_t\rvert \times IRProj$	-0.003	-0.002	-0.002	0.001	-0.001	0.002	0.001	-0.001
	(0.00)	(0.00)	(0.00)	(0.00)	(0.00)	(0.00)	(0.00)	(0.00)
$\lvert\epsilon_t\rvert \times DualProj$	-0.001	0.001	-0.001	0.001	-0.000	-0.000	0.003*	-0.001
	(0.00)	(0.00)	(0.00)	(0.00)	(0.00)	(0.00)	(0.00)	(0.00)
$\lvert\epsilon_t\rvert \times ADProj$	-0.001	0.001	0.001	0.002	-0.002	-0.002	0.004**	-0.002
	(0.00)	(0.00)	(0.00)	(0.00)	(0.00)	(0.00)	(0.00)	(0.00)
$\Delta E_{t-1}\pi_t = 0$			0.493***	0.314***			0.892***	0.624***
			(0.15)	(0.11)			(0.10)	(0.12)
$\lvert E_{t-2}\pi_{t-1} - \pi_{t-1}\rvert$			-0.005*	-0.012***			-0.013***	-0.007***
			(0.00)	(0.00)			(0.00)	(0.00)
α	-1.445***	-1.506***	-1.270***	-0.976***	-1.784***	-1.515***	-1.061***	-1.219***
	(0.14)	(0.15)	(0.15)	(0.13)	(0.16)	(0.16)	(0.13)	(0.14)
N	1619	3235	1619	3235	2433	2431	2433	2431
χ^2	21.45	68.79	33.78	58.87	92.13	46.87	140.7	67.27

(I) This table presents results from a series of mixed effects probit regressions. The dependent variables are binary variables that take the value of 1 if a subject keeps its previous forecast for the current round, and zero otherwise. IRProj, DualProj, and ADProj are treatment-specific dummies indicating the interest rate, rational dual projection, and adaptive dual projection treatments. $|E_{t-2}x_{t-1} - x_{t-1}|$ and $|E_{t-2}\pi_{t-1} - \pi_{t-1}|$ denote a subject's past forecast errors, and α denotes the estimated constant for the NoComm treatment. Robust standard errors are employed. $*p < 0.10$, $**p < 0.05$, and $***p < 0.01$.

Figure 6: Distribution of forecast updating behavior

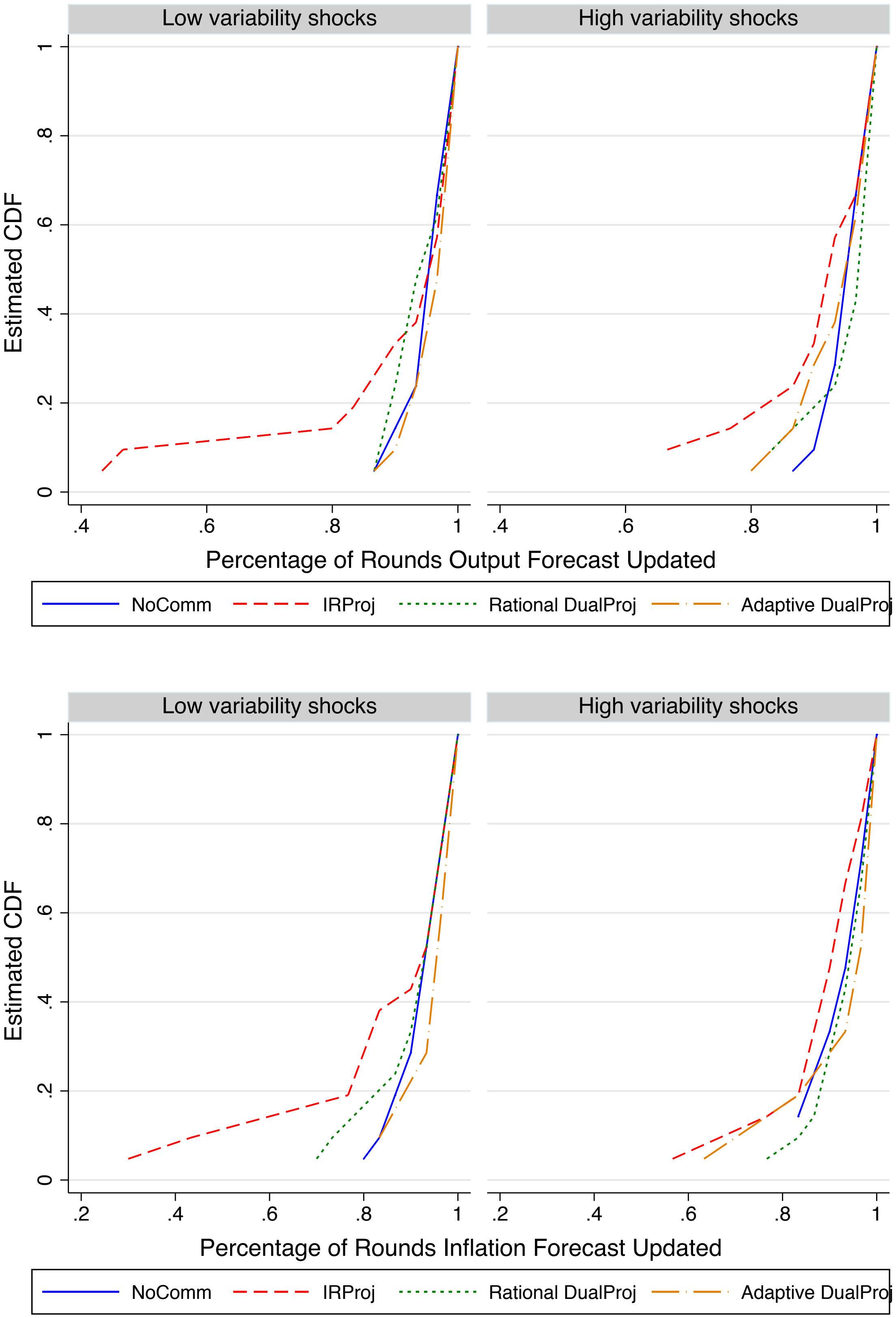

References

1. Eusepi, S. and B. Preston (2010). Central Bank Communication and Expectations Stabilization. American Economic Journal: Macroeconomics, 2(3), 235-71.

2. Evans, George W. and Honkapohja Seppo (2001). Learning and Expectations in Macroeconomics. Princeton University Press.

3. Ferrero, G., and A. Secchi (2010). Central Banks' Macroeconomic Projections and Learning. Bank of Italy Working Paper No. 782.

4. Holt, C. A. (1986). Preference reversals and the independence axiom.The American Economic Review,76(3), 508-515.

5. Karni, E. and Safra, Z. (1987). " Preference reversal" and the observability of preferences by experimental methods.Econometrica, 675-685.

6. Mankiw, N. G. and R. Reis (2002). Sticky information versus sticky prices: a proposal to replace the New Keynesian Phillips curve. The Quarterly Journal of Economics, 117(4), 1295-1328.

7. Marcet, Albert and Sargent, Thomas J. (1989). Convergence of Least-Squares Learning in Environments with Hidden State Variables and Private Information. Journal of Political Economy, 97(6), 1306-1322.

8. Reis, R. (2006). Inattentive consumers. Journal of Monetary Economics, 53(8), 1761-1800.
